# Identification of a candidate PRODH allosteric inhibitor MBTP-15324: modulation of proline metabolite profiles and ECM-associated gene-expression changes in lung cancer cells

**DOI:** 10.1080/14756366.2026.2719719

**Published:** 2026-08-24

**Authors:** Yuxuan Zhou, Dingyuan Bai, Zhihan Li, Ciqin Li, Chunqiong Li, Mengyao Chen, Cheng Guo, Jun Yan, Yonglong Han, Quanjun Yang

**Affiliations:** ^a^ College of Food Science and Technology, Shanghai Ocean University, Shanghai, China; ^b^ Department of Pharmacy, Shanghai Sixth People’s Hospital Affiliated Shanghai Jiao Tong University School of Medicine, Shanghai, China; ^c^ School of Biomedical Engineering, Donghua University, Shanghai, China; ^d^ School of Life Sciences, Beijing University of Chinese Medicine, Beijing, China; ^e^ Institute of Molecular Medicine, College of Future Technology, Peking University, Beijing, China

**Keywords:** Proline dehydrogenase (PRODH), candidate allosteric inhibitor, lung cancer, proline metabolism, extracellular matrix (ECM)

## Abstract

Current proline dehydrogenase (PRODH) inhibitors lack scaffold diversity. Through virtual screening, microscale thermophoresis, and molecular dynamics simulations, we identified MBTP-15324 as a candidate novel PRODH allosteric inhibitor (Kd = 1.45 μM). In PRODH-overexpressing lung cancer cells (A549 and LLC), MBTP-15324 significantly attenuated cell viability, colony formation, and migration. Furthermore, multi-omics analyses revealed that this putative inhibitor partially reversed arginine-proline metabolic dysregulation and downregulated transcriptional programs associated with inflammation, cell adhesion, and the extracellular matrix (ECM). In conclusion, MBTP-15324 provides a novel-scaffold chemical starting point for future PRODH-targeted therapies and metabolic interventions in lung cancer.

## HIGHLIGHTS

1. MBTP-15324 is identified as a candidate PRODH allosteric inhibitor.
2. MBTP-15324 exhibits low-micromolar binding affinity within the PRODH-GFP system.
3. Molecular dynamics simulations support its stable binding within the predicted Cavity1 pocket of PRODH.
4. MBTP-15324 preferentially inhibits proliferation and migration-related phenotypes in PRODH-overexpressing lung cancer cells.
5. MBTP-15324 partially reverses arginine-proline metabolic dysregulation and modulates ECM-associated gene-expression changes.

## GRAPHICAL ABSTRACT

**Figure:**
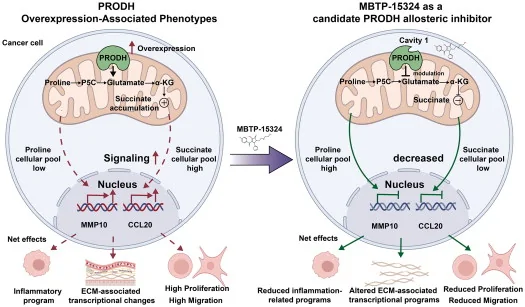

## Introduction

Lung cancer is a malignancy characterised by extremely high global incidence and mortality rates. Although targeted interventions and immunotherapy have achieved significant clinical progress in recent years, the high frequency of tumour recurrence and the rapid development of drug resistance remain the core bottlenecks limiting long-term patient survival^1^^,^^2^. In harsh microenvironments characterised by hypoxia and nutrient deprivation, tumour cells acquire survival and proliferative advantages through systemic metabolic reprogramming, which is recognised as a core hallmark of cancer development and progression^3^^,^^4^. Therefore, deeply elucidating specific metabolic vulnerabilities of malignancies and developing precise metabolic blockade strategies are emerging as highly promising frontiers in anti-tumour therapy^5^^,^^6^.

Among numerous aberrantly active metabolic networks, the remodelling of amino acid metabolism is particularly critical. Proline not only participates in protein synthesis but also serves as a crucial metabolic node connecting cellular redox homeostasis, mitochondrial metabolism, and ECM assembly^7^. Proline dehydrogenase (PRODH), a flavin-dependent oxidoreductase localised to the inner mitochondrial membrane, is the key enzyme catalysing the degradation of proline into Δ1-pyrroline-5-carboxylate (P5C)^8^. It is worth noting that the role of PRODH in tumours is context-dependent: while some studies describe it as a p53-associated tumour suppressor or pro-apoptotic factor, PRODH may also support the metabolic plasticity and malignant phenotypes of tumour cells under nutrient stress, metastatic adaptation, or specific metabolic contexts^9–12^. Thus, implementing chemical interventions targeting PRODH-overexpressing or PRODH-dependent lung cancer models holds clear biological exploratory value.

Currently, chemical interventions against PRODH predominantly focus on its orthosteric substrate-binding site, with representative molecules largely being proline analogs or mechanism-based inhibitors^13–15^. Because this region is closely associated with substrate recognition, the structural diversity, cellular permeability, and selectivity optimisation of these compounds may be limited. Therefore, exploring potential allosteric pockets on the PRODH surface and discovering novel binding scaffolds from large-scale small-molecule libraries—without directly competing for substrate binding—can provide a complementary strategy for the development of PRODH inhibitors^14^^,^^16^.

Building upon this background, the present study adopted a strategy integrating pocket prediction, virtual screening, binding validation, cellular phenotyping, and omics-based mechanistic analysis to explore PRODH allosteric inhibitors^17^^,^^18^. Given the current lack of a high-resolution experimental structure for full-length human PRODH suitable for large-scale docking, this study utilised the *Escherichia coli* PutA PRODH domain (PDB ID: 1TIW) as a bacterial surrogate structural model for pocket analysis, and the limitations of this model are explicitly acknowledged herein^19^. Subsequently, we screened for candidate allosteric pockets using CavityPlus/CorrSite. By combining virtual screening of the PMET library, microscale thermophoresis (MST), molecular dynamics (MD) simulations, PRODH-overexpressing A549 and LLC cell models, RNA-seq, and targeted metabolomics, we evaluated the relationship between the candidate PRODH allosteric inhibitor MBTP-15324, PRODH-associated phenotypes, and the metabolic network^20^.

## Results

### Prediction of PRODH allosteric pockets and hit screening

**Figure 1.**
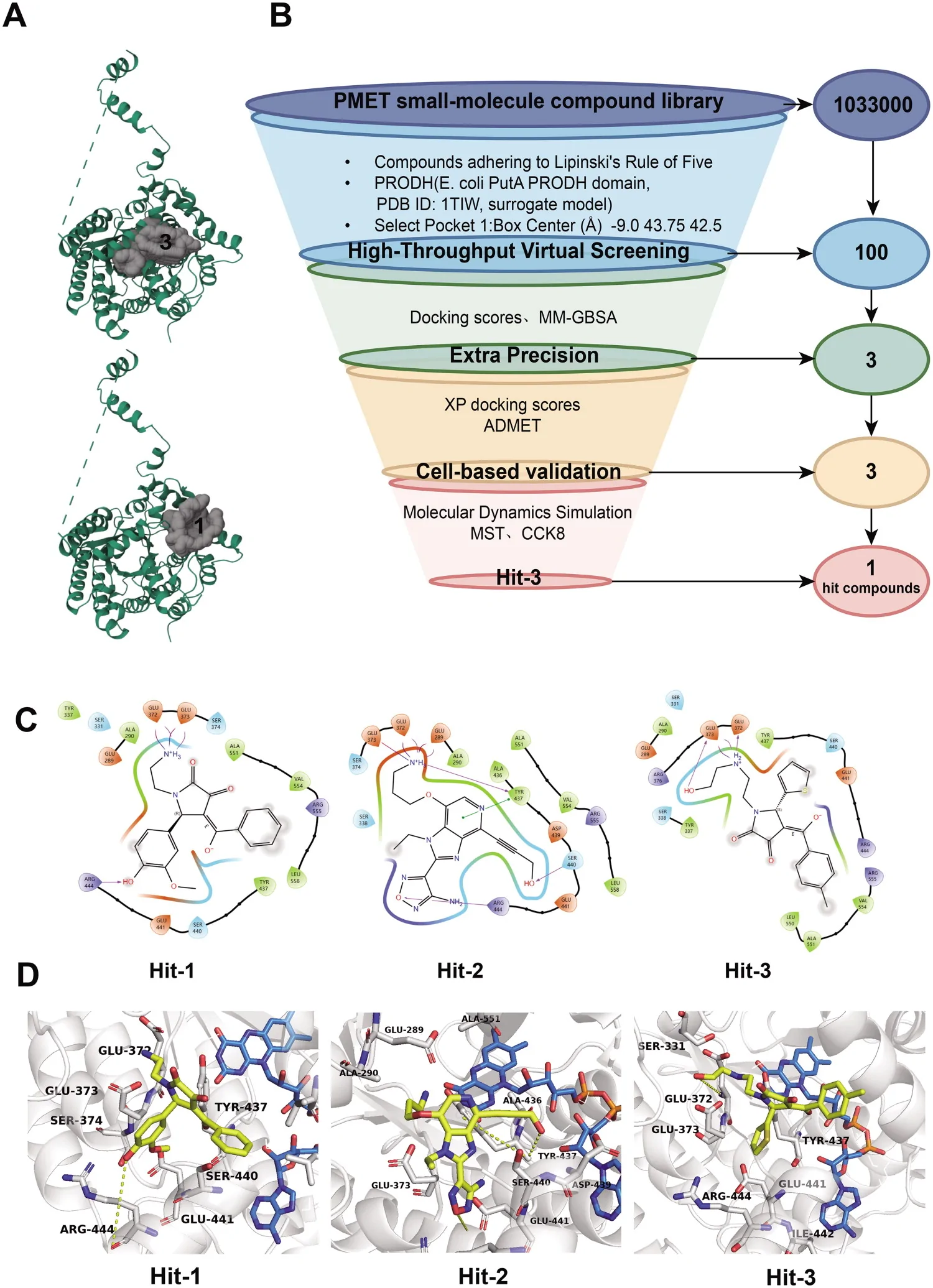
High-throughput virtual screening workflow for candidate PRODH allosteric inhibitors and molecular docking modes of candidate compounds. (A) 3D structural model of the PRODH protein (PDB ID: 1TIW). The gray surface areas denote the traditional orthosteric active site (Cavity3) and the novel allosteric pocket (Cavity1) predicted and targeted in this study. (B) Flowchart of the hierarchical high-throughput virtual screening strategy. Initial screening targeted the PMET compound library, which contains 1,033,000 small molecules. Successive filtering via Lipinski’s Rule of Five, Cavity1 grid generation, and high-throughput virtual screening (HTVS) yielded the top 100 molecules. Subsequent extra-precision (XP) flexible docking and MM-GBSA binding free energy calculations narrowed the pool to 3 candidate molecules. Finally, integrating molecular dynamics (MD) simulations, microscale thermophoresis (MST), and *in vitro* cellular validation identified Hit-3/MBTP-15324 as a candidate PRODH allosteric inhibitor. (C) 2D interaction modes of the 3 candidate compounds (Hit-1, Hit-2, Hit-3) with key amino acid residues in the Cavity1 pocket of PRODH. Different coloured backgrounds and lines represent hydrogen bonds, hydrophobic interactions, and polar networks between the ligands and the receptor. (D) Best 3D binding conformations of the 3 candidate compounds in the Cavity1 pocket. The yellow highlighted molecules represent the docked candidate ligands (Hit1-3), and the blue molecules represent the cofactor (FAD). The surrounding gray stick models indicate key interacting amino acid residues (e.g., GLU-372, TYR-437, ARG-444), and yellow dashed lines denote hydrogen bonds formed between the ligands and protein residues. (Abbreviations: PRODH, proline dehydrogenase; HTVS, high-throughput virtual screening; XP, extra precision docking; MD, molecular dynamics; MST, microscale thermophoresis; ADMET, absorption, distribution, metabolism, excretion, and toxicity.)

To systematically explore potential ligand-binding pockets on the surface of PRODH, this study utilised the CavityPlus server to perform a surface scan of the *Escherichia coli* PutA PRODH domain (PDB ID: 1TIW). It is important to emphasise that 1TIW is not the full-length human PRODH structure; rather, it serves as a bacterial surrogate structural model for PRODH pocket analysis in this study. The calculations identified multiple cavities with ligand-binding potential (Supplementary Figure 1). Among them, Cavity3 was designated as the orthosteric reference site, possessing a DrugScore of 1043.00, a surface area of 1038.25 Å^2^, and a volume of 1092.00 Å³. It is located in close proximity to key catalysis-related residues, including MET-516, TYR-552, and ARG-458 (Figure 1A).

Subsequently, the CorrSite module was employed to evaluate the allosteric correlation between the other pockets and the orthosteric site, using Cavity3 as the orthosteric benchmark. Cavity4 exhibited the highest CorrSite score (1.74); however, its negative DrugScore indicated that its druggability and dockability might be insufficient. Although Cavity2 possessed higher CorrSite and DrugScores, Cavity1 was deemed more suitable as the pocket for subsequent screening in terms of docking grid accessibility, pocket openness, and spatial accommodation for small molecules. Consequently, Cavity1 was prioritised for virtual screening in this study (Table 1).

**Table 1.** Cavity-related data.

Cavity	PredMaxpKd	PredAvepKd	DrugScore	CorrSiteScore
Cavity1	11.39	6.99	617	1.38
Cavity2	11.64	6.88	753	1.65
Cavity3	11.45	6.54	1043	**-**
Cavity4	8.87	5.66	−784	1.74
Cavity5	8.72	5.61	−371	0.87
Cavity6	6.96	5	−518	1.6
Cavity7	6.72	4.92	−1155	0.87

To search for PRODH allosteric inhibitors with novel scaffold characteristics, a hierarchical virtual screening workflow was performed. After the initial filtration of the PMET (Proline Metabolism) compound library using Lipinski’s Rule of Five and High-Throughput Virtual Screening (HTVS), the screening pool was narrowed down to high-scoring candidate molecules. Following Extra Precision (XP) docking, MM-GBSA binding free energy evaluation, and preliminary physicochemical/ADMET prediction using QikProp, three candidate molecules were ultimately obtained (Tables 2 and 3). Taking into consideration the docking scores, preliminary ADMET predictions, subsequent MST apparent binding results, and cellular phenotypic data, Hit-3 was selected as the primary putative PRODH allosteric inhibitor and designated as MBTP-15324 (Figure 1).

**Table 2.** Docking scores and ADMET data of the selected molecules.

Compound	Dockingscore	MMGBSAdGBind	Molecular weight (MW)	Hydrogen bond donors (HBD)	Hydrogen bond acceptors (HBA)	Polar surface area (PSA)*	Human oral absorption category (QikProp)	Rule of Five violations
Hit1	−7.428	−24.58	368.39	3.000	7.250	129.944	2	0
Hit2	−7.886	−40.80	357.37	5.000	8.950	148.172	2	0
Hit3	−7.270	−25.29	386.47	2.000	7.950	109.013	3	0

**Table 3.** Evaluation of other ADMET properties of the selected molecules.

Compound	QPlogPo/w	QPlogS	QPlogHERG	QPlogBB	QPPCaco	QPPMDCK	QPlogKhsa
Hit1	1.480	−3.658	−6.366	−1.665	20.808	8.326	−0.040
Hit2	−0.208	−2.646	−5.993	−2.627	4.754	1.688	−0.515
Hit3	2.265	−3.741	−6.799	−1.300	61.663	38.337	0.007

### MST assay reveals low-micromolar binding affinity of MBTP-15324 within the PRODH-GFP fusion protein system

To quantitatively evaluate the binding affinities of the candidate compounds to PRODH, microscale thermophoresis (MST) was employed to measure the interactions between the candidate small molecules and the PRODH-GFP fusion protein cell lysate system. The measurements were performed using a NanoTemper Monolith NT.115 instrument, and data were analysed with MO.Affinity Analysis v2.3 software. The detection temperature was set to 25.0 °C, utilising Nano-BLUE excitation with an excitation power of 20% and an MST power of 40%. Preliminary tests indicated no obvious capillary adsorption or sample aggregation. Based on fluorescence intensity, the PRODH-GFP lysates and GFP-only control lysates were diluted 1:5 and 1:10, respectively.

**Figure 2.**
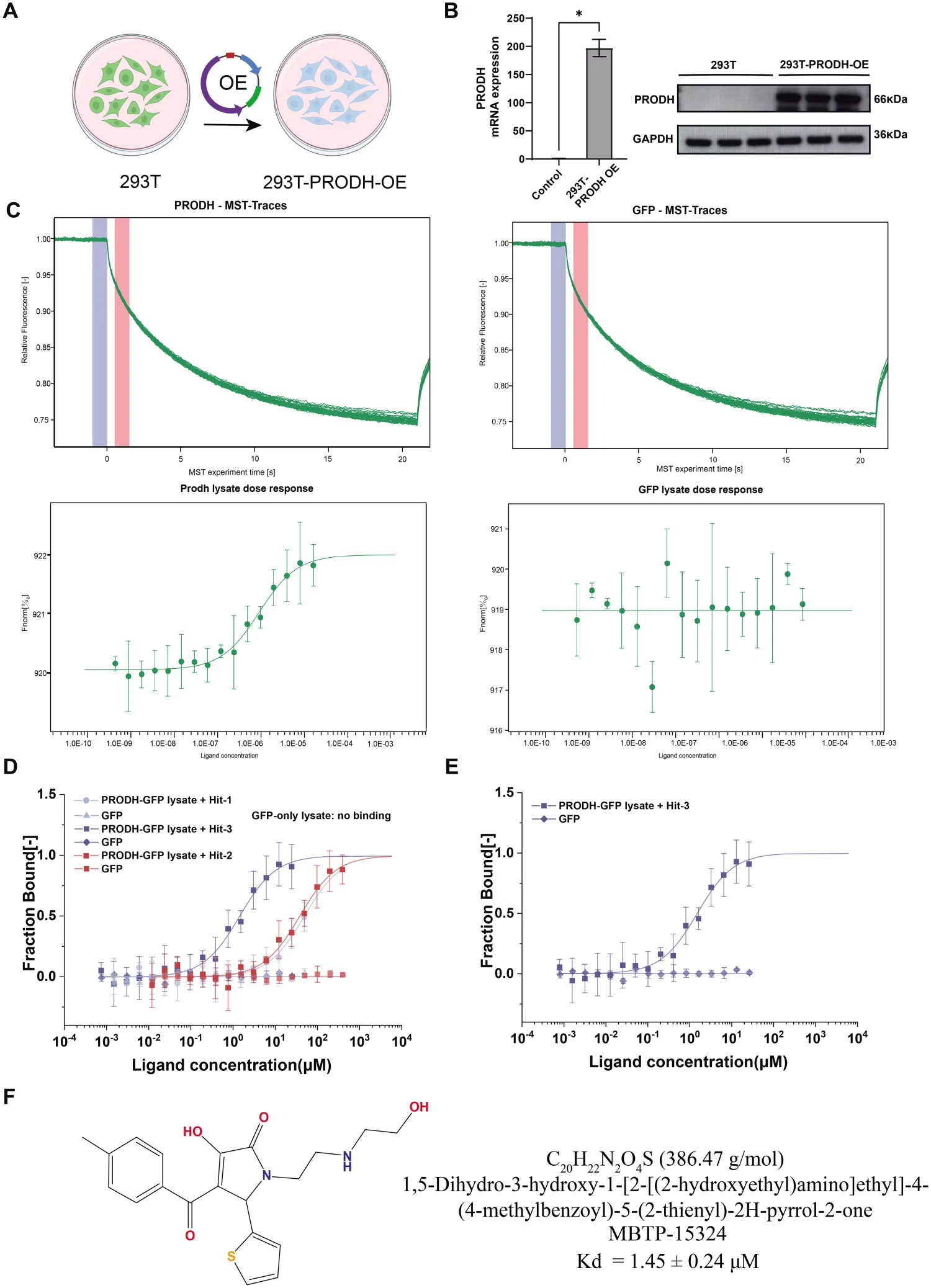
MST binding affinity assay of MBTP-15324 with the PRODH-GFP fusion protein system. (A) Fluorescence microscopy images showing the fluorescence signals of 293T cells expressing the PRODH-GFP fusion protein and control cells. (B) RT-qPCR and Western blot validation of PRODH expression. (C) Raw MST fluorescence traces and the GFP-only tag control. No detectable binding signal was observed in the GFP-only control, suggesting that the MST response is unlikely to originate from the GFP tag itself. (D) MST fitting curves and Kd values for Hit-1 and Hit-2 with the PRODH-GFP lysate system. (E) Quantitative MST analysis of Hit-3/MBTP-15324 with the PRODH-GFP lysate system. The fitting curve exhibits a concentration-dependent sigmoidal response, with Kd = 1.45 ± 0.24 μM and a Signal/Noise = 22.98. (F) 2D chemical structure, molecular formula (C_20_H_22_N_2_O_4_S), and molecular weight (386.47 g/mol) of MBTP-15324.

In the affinity assays, all candidate compounds generated fittable MST (Table 4) responses with the PRODH-GFP system. Among them, Hit-3 exhibited the strongest binding signal, presenting a clear concentration-dependent sigmoidal fitting curve within the concentration range of 25 μM to 0.000763 μM. Its Kd was 1.4495 × 10^-6 M (approximately 1.45 ± 0.24 μM), and the signal-to-noise ratio was 22.98, meeting the criteria for high-quality binding data. In contrast, the Kd values for Hit-2 and Hit-1 were approximately 49.8 μM and 38.7 μM, respectively, indicating weaker affinities than Hit-3. No fittable binding signal was observed in the GFP-only control group, suggesting that these MST responses are unlikely to originate from non-specific interactions between the candidate compounds and the GFP tag itself. Based on the methyl benzoyl-thienyl-pyrrolone core scaffold of this compound, Hit-3 was designated as MBTP-15324 (Figure 2).

**Table 4.** Results of MST affinity assays.

Compound	Fluorescent target system	Ligand range	Kd	Kd confidence	Signal/Noise
Hit-1	PRODH-GFP lysate	400–0.0122 μM	38.7 μM	±8.30 μM	19.10
Hit-2	PRODH-GFP lysate	50–0.00153 μM	49.8 μM	±23.8 μM	30.80
Hit-3/MBTP-15324	PRODH-GFP lysate	25–0.000763 μM	1.45 μM	±0.24 μM	22.98
GFP control	GFP-only lysate	matched ligand ranges	No binding	/	0

*Note*: The Kd values were fitted using MO.Affinity Analysis v2.3 software based on the MST assay results. The GFP-only lysate served as the tag background control, and no fittable binding signal was observed, suggesting that the aforementioned MST responses are unlikely to arise from non-specific interactions between the compounds and the GFP tag itself. Because the experiment utilised a PRODH-GFP fusion protein system derived from cell lysates rather than purified recombinant PRODH protein, the Kd values are defined as apparent affinity parameters within the lysate environment.

### All-Atom molecular dynamics (MD) simulations support the binding stability of MBTP-15324 within the predicted cavity1 pocket

To further evaluate the binding stability of the MBTP-15324 and PRODH protein complex, this study conducted a 100-ns molecular dynamics (MD) simulation of the PRODH–FAD–MBTP-15324 complex and analysed metrics including RMSD, RMSF, Rg, hydrogen bonds, SASA, and protein-ligand interaction energies.

**Figure 3.**
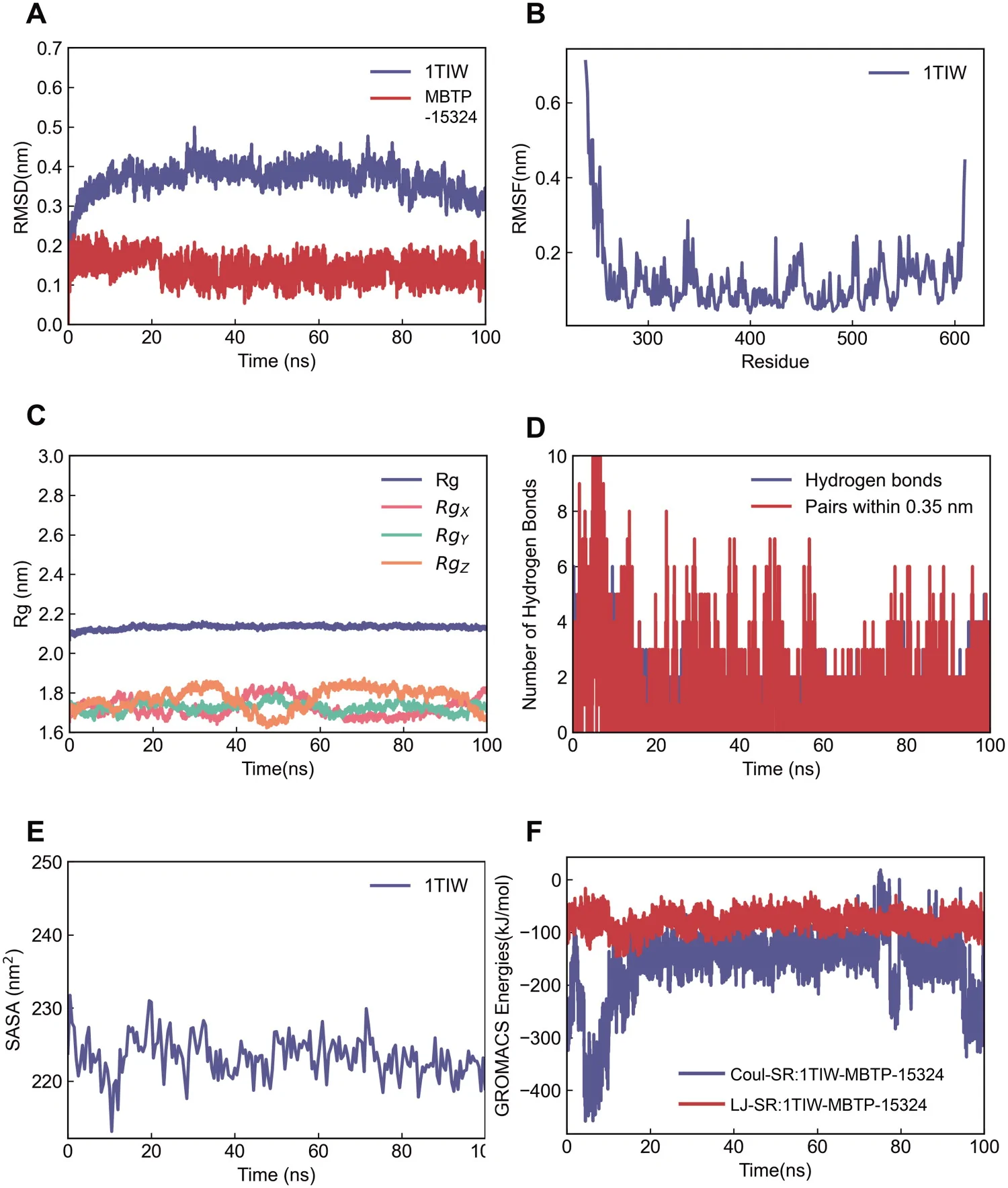
100-ns all-atom molecular dynamics (MD) simulations reveal the binding stability of MBTP-15324 within the PRODH allosteric pocket. (A) Trajectory of the root mean square deviation (RMSD) of the PRODH protein backbone (1TIW, blue curve) and the ligand molecule (MBTP-15324, red curve) over the 100-ns simulation. This panel reflects the overall conformational stability of the complex during the simulation. (B) Root mean square fluctuation (RMSF) chart of individual amino acid residues of the PRODH protein during the simulation, used to evaluate the flexibility of the local protein structure in the bound state. (C) Radius of gyration (Rg, dark blue curve) of the protein complex and its components along the X, Y, and Z spatial directions (Rgx, Rgy, Rgz) over time, indicating the compactness of the overall protein conformation and the absence of abnormal folding/unfolding. (D) Analysis of intermolecular interactions formed between the MBTP-15324 ligand and the PRODH binding pocket during the simulation. The blue curve represents the number of hydrogen bonds, and the red curve represents the number of contact pairs within 0.35 nm. (E) Trajectory of the solvent-accessible surface area (SASA) of the complex over the simulation time, reflecting the stability of the protein’s surface exposure in the solvent. (F) Short-range non-bonded interaction energies between PRODH (1TIW) and the ligand (MBTP-15324) over time. The blue curve represents the short-range Coulombic electrostatic interaction energy (Coul-SR), and the red curve represents the short-range Lennard-Jones van der Waals interaction energy (LJ-SR). (Abbreviations: MD, molecular dynamics; RMSD, root mean square deviation; RMSF, root mean square fluctuation; Rg, radius of gyration; SASA, solvent-accessible surface area; Coul-SR, short-range Coulombic interaction energy; LJ-SR, short-range Lennard-Jones interaction energy.)

The RMSD results revealed that the protein backbone exhibited some fluctuations during the initial phase of the simulation but gradually stabilised after approximately 10 ns, largely maintaining a range of 0.30–0.45 nm. The RMSD of the ligand MBTP-15324 was primarily distributed within the 0.10–0.20 nm range, showing no continuous upward trend or obvious detachment from the binding pocket, indicating that the overall conformation of the complex was stable (Figure 3A). RMSF analysis further demonstrated that most protein residues exhibited low fluctuations; the higher fluctuations were mainly concentrated at the termini and local loop regions, whereas the regions associated with the binding pocket remained relatively stable overall (Figure 3B).

Radius of gyration (Rg) analysis showed that the overall Rg of the protein remained essentially stable throughout the 100-ns simulation, with no obvious structural loosening or abnormal unfolding observed (Figure 3C). Hydrogen bond and short-range contact analyses indicated that MBTP-15324 continuously formed hydrogen bonds and short-range contacts with pocket residues during the simulation, suggesting a relatively stable interaction network within the binding site (Figure 3D). The SASA curve showed minimal overall changes, indicating that the solvent-accessible surface area of the complex did not change significantly during the simulation (Figure 3E).

Further analysis of protein-ligand interaction energies revealed that the short-range Coulombic interaction energy (Coul-SR) and Lennard-Jones interaction energy (LJ-SR) generally remained negative during the simulation. This suggests the presence of sustained, favourable non-bonded interactions between MBTP-15324 and PRODH, among which van der Waals and hydrophobic interactions likely made critical contributions to maintaining the stable binding of the ligand (Figure 3F).

In summary, the 100-ns MD simulation results support that the PRODH–FAD–MBTP-15324 complex remains relatively stable over the simulation timescale, and MBTP-15324 can maintain a stable binding conformation within the predicted Cavity1 pocket.

### MBTP-15324 attenuates proliferation, colony formation, and 2D migration-related phenotypes in PRODH-overexpressing lung cancer cells

**Figure 4.**
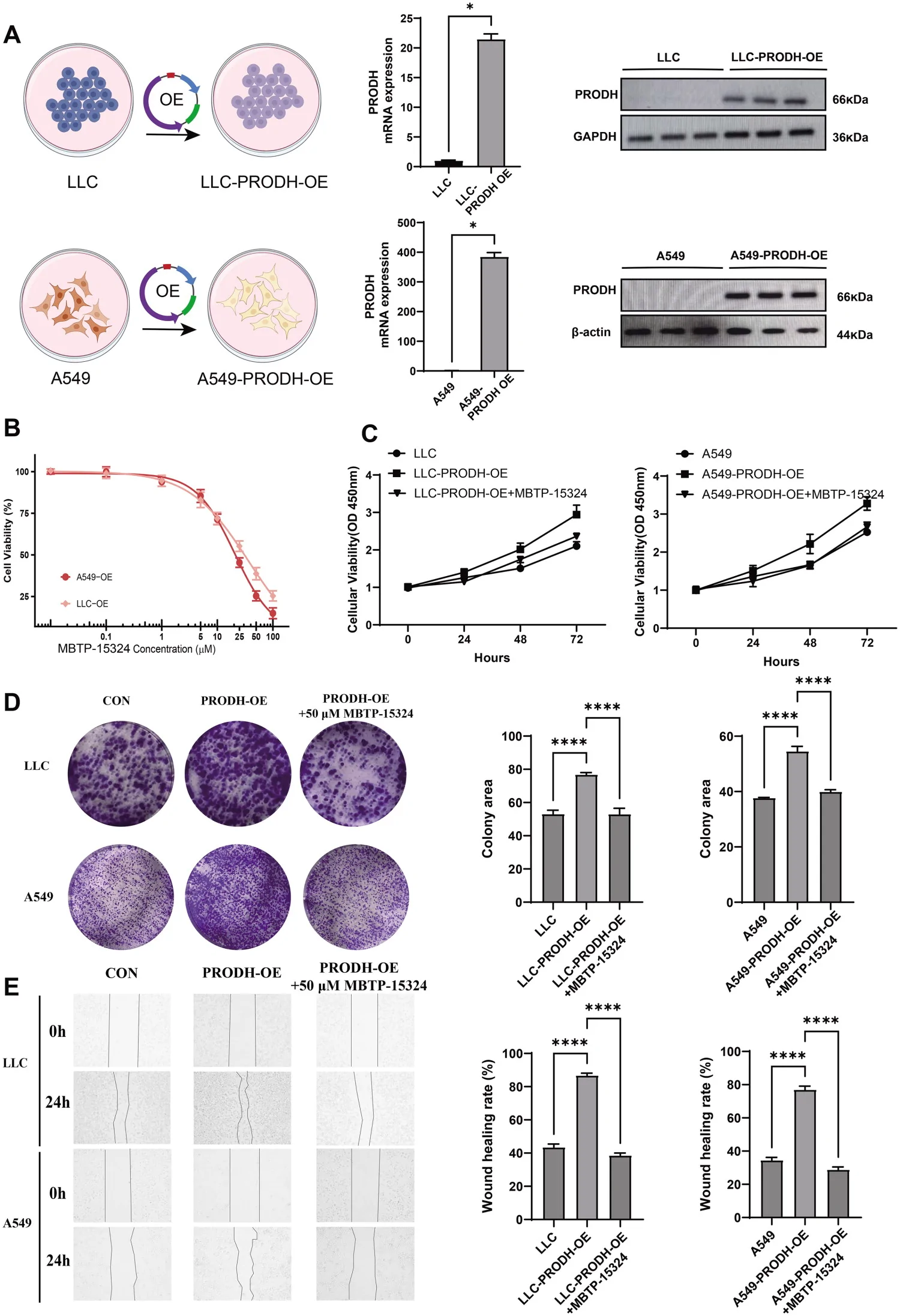
MBTP-15324 preferentially inhibits proliferation, colony formation, and 2D migration-related phenotypes in PRODH-overexpressing lung cancer cells. (A) Construction and validation of stable PRODH-overexpressing murine Lewis lung carcinoma (LLC) and human non-small cell lung cancer (A549) cell models (PRODH-OE). Left: Schematic illustration of the stable cell line construction process. Middle: Relative *PRODH* mRNA expression levels detected by RT-qPCR (*n* = 3). Right: Gel images of Western blot analysis showing the expression abundance of PRODH protein (66 kDa) and internal controls (GAPDH, 36 kDa or β-actin, 44 kDa). (B) Effects of gradient concentrations (0.1-100 μM) of MBTP-15324 on cell viability assessed by the CCK-8 assay. Dose-response curves indicate that PRODH-OE cells are sensitive to MBTP-15324 intervention (*n* = 3). (C) Time-dependent cell proliferation curves. Trajectories of cell viability changes in LLC (left) and A549 (right) cells following continuous treatment with 50 μM MBTP-15324 for 0, 24, 48, and 72 hours, assessed by the CCK-8 assay (*n* = 3). (D) Colony formation assay evaluating the clonogenic proliferative capacity of the cells. Left: Representative images of crystal violet-stained cell colonies after 10–14 days of treatment with 50 μM MBTP-15324. Right: Bar graphs of the relative colony formation area quantified using ImageJ software (*n* = 3). (E) 2D wound healing assay evaluating the directed migration potential of the cells. Left: Representative microscopic field images at 0 and 24 hours of 50 μM MBTP-15324 treatment. Right: Bar graphs showing the quantitative analysis of the wound healing rate (*n* = 3). (Data expression and statistical notes: All quantitative data are presented as mean ± SEM, with 3 independent biological replicates per group (*n* = 3). Comparisons between two groups were conducted using an independent samples t-test, and comparisons among multiple groups were analysed using one-way ANOVA. *p < 0.05, ****p < 0.0001. Abbreviations: PRODH, proline dehydrogenase; OE, overexpression; LLC, murine Lewis lung carcinoma cells; A549, human non-small cell lung cancer cells.)

To translate the *in vitro* target affinity of MBTP-15324 into anti-tumour biological efficacy, this study systematically evaluated its intervention effects on the malignant phenotypes of lung cancer across multidimensional cellular models. First, we established stable PRODH-overexpressing (PRODH-OE) cell models in human non-small cell lung cancer (A549) and murine Lewis lung carcinoma (LLC) cell lines (Figure 4A). RT-qPCR analysis indicated that the transcriptional levels of the target gene in the LLC and A549 overexpression lines were significantly upregulated compared to the wild-type (WT). Western blot results further confirmed the high-level expression of the PRODH protein at 66 kDa, verifying the successful construction of the genetic models.

Based on this model, the CCK-8 assay was utilised to evaluate the cell proliferation inhibitory effect of MBTP-15324. The dose-response curves (Figure 4B) showed that when MBTP-15324 was applied to PRODH-overexpressing cells, the IC50 values for the OE groups were 21.36 μM and 32.84 μM, respectively. Based on the dose-response curves and the subsequent phenotypic observation window, 50 μM was selected for the colony formation and wound healing assays. Considering that this concentration is higher than the apparent IC50 range of the PRODH-OE cells, we defined it as the cellular phenotypic intervention concentration. This decision was interpreted comprehensively alongside the clonogenic, migratory, and omics results, rather than interpreting the CCK-8 readouts solely as specific enzyme inhibitory effects. Time-dependent cell viability assays (Figure 4C) further demonstrated that treatment with 50 μM MBTP-15324 blunted the cell viability advantage associated with PRODH overexpression. Given that CCK-8 readouts can be influenced by the mitochondrial redox state, this study utilised them as preliminary cell viability screening data, combined with colony formation and wound healing assays to collaboratively evaluate cellular phenotypes.

In assays evaluating malignant progression phenotypes, clonogenic analysis (Figure 4D) confirmed that PRODH overexpression significantly enhanced the clonogenicity of LLC and A549 cells, with the clone colony area showing a statistically significant expansion compared to the control group. Following intervention with 50 μM MBTP-15324, this pro-proliferative effect was effectively blocked, and the colony formation area was significantly reduced. Furthermore, the 2D wound healing assay (Figure 4E) focused on evaluating directed cell migration capacity. At the 24-h observation endpoint, the wound healing rate of the PRODH-OE group significantly increased to approximately 80%, far exceeding the basal migration level of the control group (30%-40%). Following co-incubation with MBTP-15324, this cell migration process was markedly suppressed, with the healing rates of both cell lines dropping below 40% and 30%. Taken together, the *in vitro* cytological results suggest that MBTP-15324 preferentially inhibits the viability, colony formation, and 2D migration-related phenotypes of PRODH-overexpressing lung cancer cells.

### RNA-seq analysis indicates MBTP-15324 partially reverses PRODH overexpression-associated inflammation and ECM-related transcriptional programs in A549 cells

To systematically elucidate the molecular mechanisms by which MBTP-15324 intervenes in the PRODH-mediated malignant progression of lung cancer at the genome-wide level, this study conducted bulk RNA-seq sequencing and multi-omics analysis on the A549 cell models (Control group, PRODH-OE group, and OE+MBTP-15324 group).

**Figure 5.**
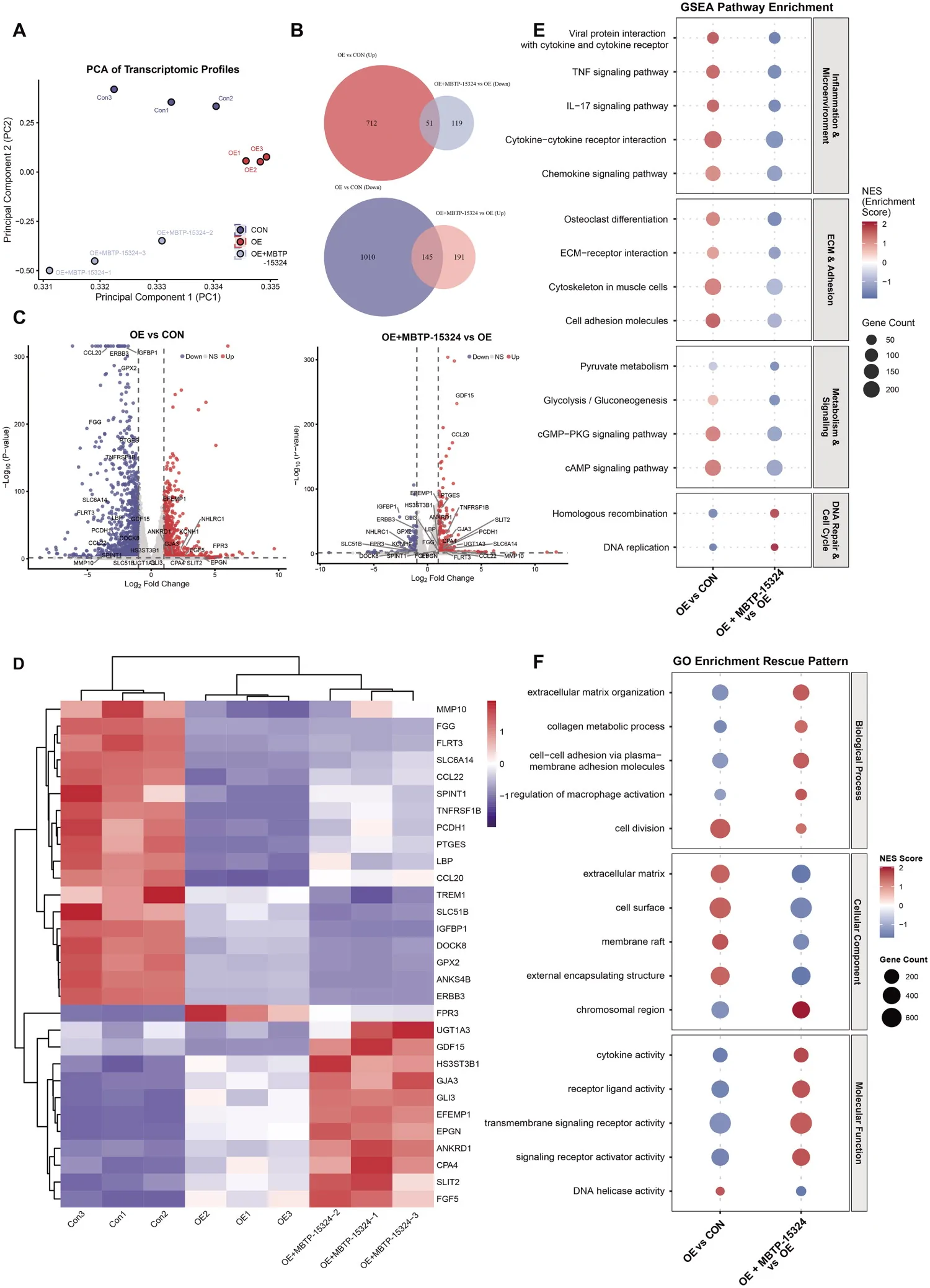
RNA-seq analysis indicates MBTP-15324 partially reverses PRODH overexpression-associated inflammation and ECM-associated gene-expression changes. (A) Principal component analysis (PCA) of cellular transcriptomic profiles. The plot shows the clustering separation and regression trajectories of the wild-type control group (CON), PRODH-overexpressing group (OE), and drug intervention group (OE+MBTP-15324) in the dimensionally reduced space (*n* = 3 per group). (B) Venn diagram of differentially expressed genes (DEGs). It displays the overlapping intersections between genes significantly upregulated/downregulated in the OE group and those significantly reverse-regulated (i.e., rescued) following MBTP-15324 intervention. (C) Volcano plots of global gene expression differences. Left: OE vs. CON; Right: OE+MBTP-15324 vs. OE. Red dots represent significantly upregulated genes (Up), dark blue dots represent significantly downregulated genes (Down), and gray dots denote non-significant genes (NS). Key genes associated with the microenvironment and matrix remodelling (e.g., *MMP10*, *CCL20*, *FLRT3*) are highlighted. (D) Hierarchical clustering heatmap of representative key DEGs. It illustrates the changes in the expression patterns of disease-associated transcripts across independent samples in each group. The colour scale represents relative gene expression abundance, with red indicating high expression and blue indicating low expression. (E) Bubble plots of Gene Set Enrichment Analysis (GSEA). The comparison reveals shifts in the enrichment status of key pathways—such as microenvironmental inflammation, ECM and adhesion, metabolism, and the cell cycle—in OE vs. CON and OE+MBTP-15324 vs. OE. (F) Bubble plot of the rescue pattern in Gene Ontology (GO) enrichment. It systematically displays the reversing effects of MBTP-15324 intervention on PRODH-induced aberrant changes in biological processes (BP, e.g., ECM organisation, collagen metabolic process), cellular components (CC), and molecular functions (MF). (Data expression and abbreviations: RNA-seq included 3 independent biological replicate samples per group (n = 3). The strict screening threshold for DEGs was set at |log_2_FC| > 1 and P.adj < 0.05. In panels (E) and (F), dot colour represents the Normalised Enrichment Score (NES), where red indicates pathway activation or positive enrichment, and blue indicates pathway suppression or negative enrichment; dot size is proportional to the number of DEGs included in the pathway (Gene Count). Abbreviations: CON, control group; OE, PRODH overexpression group; PCA, principal component analysis; GSEA, gene set enrichment analysis; NES, normalised enrichment score; GO, gene ontology.)

Principal Component Analysis (PCA) (Figure 5A) showed that the three groups of samples exhibited a separation trend in the dimension-reduced space. PRODH overexpression shifted the overall transcriptomic profile of the cells away from the control group; following MBTP-15324 intervention, the transcriptomic profile of the OE+MBTP-15324 group shifted back towards the control group, suggesting that the compound can partially reverse the transcriptional alterations associated with PRODH overexpression. Differential expression analysis further revealed that PRODH overexpression induced the upregulation of multiple genes related to inflammatory signalling, matrix degradation, and cell adhesion, including *MMP10*, *CCL20*, *CCL22*, and *FLRT3*. After MBTP-15324 treatment, the expression of these transcripts showed a downward trend (Figure 5B-D).

Gene Set Enrichment Analysis (GSEA) and Gene Ontology (GO) analysis (Figure 5E-F) demonstrated that PRODH overexpression was enriched for pathways such as TNF signalling, IL-17 signalling, cytokine-receptor interactions, ECM-receptor interactions, and cell adhesion molecules. Following MBTP-15324 treatment, the enrichment direction of these pathways was reversed or weakened, indicating its capacity to modulate the inflammation- and ECM-associated gene-expression changes associated with PRODH overexpression. Because the *in vitro* monoculture cell model of A549 cannot completely mimic the tumour immune microenvironment, this study cautiously describes these results as alterations in inflammation-related and ECM-related transcriptional programs.

In summary, the RNA-seq results suggest that PRODH overexpression not only alters the expression of metabolism-related genes, but also drives transcriptional programs associated with inflammation, cell adhesion, and ECM-associated gene regulation. MBTP-15324 can partially reverse these transcriptional alterations in A549 PRODH-OE cells, which is consistent with its effects of attenuating clonogenic and 2D migration-related phenotypes (Supplementary Figure 2,3).

Building on these transcriptomic findings, PRODH overexpression may perturb intracellular amino acid metabolic homeostasis by regulating the expression of key metabolic enzymes, in addition to mediating aberrant inflammation- and ECM-related transcription. To directly verify the proline metabolic dysregulation induced by PRODH overexpression and the intervention effect of MBTP-15324 at the metabolite level, and to match transcriptional alterations with corresponding actual metabolic phenotypes, this study further performed targeted metabolomics analysis. We quantified the abundance of metabolites in the arginine-proline metabolic axis and its upstream and downstream related pathways, and conducted integrative analysis with transcriptomic data to systematically elucidate the metabolism-transcription coupled mechanism underlying MBTP-15324-mediated modulation of malignant phenotypes in PRODH-overexpressing lung cancer cells.

### Targeted metabolomics and integrative analysis reveal MBTP-15324 modulates the PRODH overexpression-associated arginine-proline metabolic network in A549 cells

**Figure 6.**
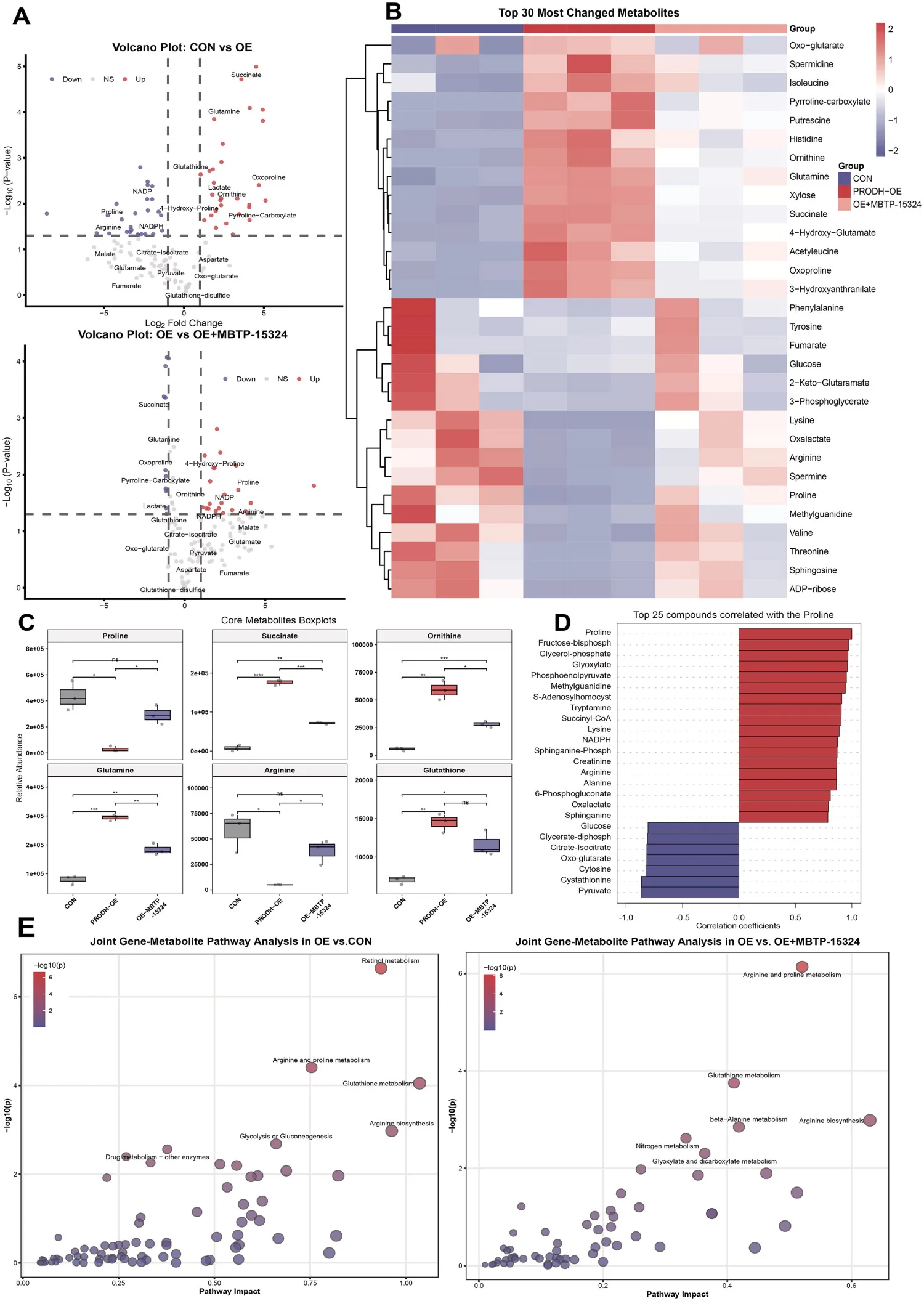
Targeted metabolomics and gene-metabolite integrative analysis indicate MBTP-15324 modulates the PRODH-associated arginine-proline metabolic network. (A) Volcano plot analysis of differential metabolites. Top: Comparison between the PRODH overexpression group (OE) and the wild-type control group (CON). Bottom: Comparison between the MBTP-15324 intervention group (OE+MBTP-15324) and the OE group. Red dots represent metabolites with significantly upregulated relative ­abundance (Up), blue dots represent significantly downregulated metabolites (Down), and gray dots indicate no significant difference (NS). Core metabolite nodes such as proline, succinate, glutamine, and ornithine are highlighted. (B) Hierarchical clustering heatmap of the Top 30 Most Changed Metabolites in relative abundance among groups. The colour scale reflects the relative abundance of metabolites in each sample, with red representing high abundance and blue representing low abundance. The results show that MBTP-15324 intervention shifts the overall metabolic profile of OE cells back towards the characteristics of the control group (CON). (C) Box plots showing the relative abundance of core metabolites (proline, succinate, ornithine, glutamine, arginine, and glutathione) across groups. It visually presents the significant reversal effects of MBTP-15324 on PRODH-mediated metabolic anomalies such as proline depletion and succinate/ornithine accumulation. (D) Bar chart of Pearson/Spearman correlations for the top 25 metabolites highly correlated with proline abundance. Red bars indicate positive correlations (correlation coefficient > 0), and blue bars indicate negative correlations (correlation coefficient < 0). (E) Bubble plots of Joint Gene-Metabolite Pathway Analysis. Left: OE vs. CON; Right: OE vs. OE+MBTP-15324. The horizontal axis represents Pathway Impact, and the vertical axis represents enrichment significance (-log10(P-value)). Bubble size and colour depth reflect the significance of pathway enrichment, highlighting that Arginine and proline metabolism and Glutathione metabolism are the core metabolic networks most affected by the intervention. (Data expression and statistical notes: All metabolomics analyses were based on 3 independent biological replicate samples (n = 3). The strict screening threshold for differential metabolites was VIP > 1 and p < 0.05. The box plots in panel C display the median and interquartile ranges of the data, with whiskers representing maximum and minimum values; comparisons among multiple groups were analysed using one-way ANOVA followed by appropriate post-hoc tests. Abbreviations: CON, negative control group; OE, PRODH overexpression group; NS, no significant statistical difference.)

To clarify the impact of PRODH overexpression and MBTP-15324 intervention on the cellular metabolic state, this study performed targeted metabolomics analysis on cells from the CON, PRODH-OE, and OE+MBTP-15324 groups. Volcano plot results showed that, compared to the control group, PRODH overexpression significantly altered the levels of various intracellular metabolites, particularly those related to proline metabolism, the tricarboxylic acid (TCA) cycle, and redox homeostasis (Figure 6A). In the PRODH-OE group, metabolites such as succinate, glutamine, glutathione, oxoproline, ornithine, and pyrroline-carboxylate were markedly elevated, whereas proline and arginine levels were reduced. Notably, proline exhibited a significant decrease, while succinate showed the most pronounced upward trend, suggesting that PRODH overexpression promotes proline catabolism and the activation of its downstream mitochondrial metabolic pathways. Following MBTP-15324 treatment, the metabolic alterations induced by PRODH overexpression were partially reversed, manifested by the recovery of proline and arginine levels, alongside decreases in metabolites such as succinate, glutathione, oxoproline, and ornithine (Figure 6A). Heatmap clustering analysis further revealed that the CON, PRODH-OE, and OE+MBTP-15324 groups possessed distinctly different metabolic profiling characteristics (Figure 6B). In the PRODH-OE group, metabolites including oxo-glutarate, pyrroline-carboxylate, ornithine, glutamine, succinate, and oxoproline were generally elevated, while proline, arginine, and spermine were reduced. Upon MBTP-15324 intervention, the overall metabolic profile of the OE+MBTP-15324 group reverted towards that of the CON group, indicating that MBTP-15324 effectively mitigates the aberrant metabolic remodelling induced by PRODH overexpression.

Further quantitative analysis of representative core metabolites revealed that PRODH overexpression significantly reduced proline levels, with its relative abundance dropping by more than 90% compared to the CON group (Figure 6C). Conversely, succinate and ornithine were markedly elevated in the PRODH-OE group, increasing by approximately 20-fold and 10-fold, respectively. Additionally, glutamine and glutathione levels also increased significantly, while arginine levels decreased. After MBTP-15324 treatment, proline and arginine levels recovered significantly, while succinate, ornithine, glutamine, and glutathione levels decreased, further demonstrating that MBTP-15324 inhibits PRODH-mediated enhancements in proline metabolism. Correlation analysis showed that proline levels were closely correlated with multiple metabolites associated with amino acid metabolism, glycolysis, and redox status (Figure 6D). Specifically, proline correlated positively with arginine, alanine, NADPH, phosphoenolpyruvate, and fructose-bisphosphate, but negatively with pyruvate, oxo-glutarate, citrate-isocitrate, and glucose. This indicates that PRODH-mediated alterations in proline metabolism not only affect the metabolite abundance of proline itself but may also be tightly coupled with changes in cellular energy metabolism and redox homeostasis. Integrated gene-metabolite pathway analysis revealed that PRODH overexpression primarily impacted pathways such as Arginine and proline metabolism, Glutathione metabolism, Arginine biosynthesis, and Glycolysis/Gluconeogenesis (Figure 6E). Among them, Arginine and proline metabolism, as well as Glutathione metabolism, exhibited high pathway impact values and statistical significance. Following MBTP-15324 treatment, Arginine and proline metabolism remained the most significantly affected pathway, while pathways like Glutathione metabolism and Nitrogen metabolism also underwent notable changes, suggesting that the effects of MBTP-15324 are largely concentrated within PRODH-associated amino acid and redox metabolic networks.

In conclusion, PRODH overexpression induced a metabolic reprogramming primarily characterised by decreased proline, elevated succinate and ornithine, and decreased arginine. MBTP-15324 intervention partially reversed these metabolic alterations, suggesting it likely blunts the enhanced proline catabolism associated with PRODH overexpression.

### MBTP-15324 partially reverses perturbations in the arginine-proline metabolic pathway associated with PRODH overexpression

**Figure 7.**
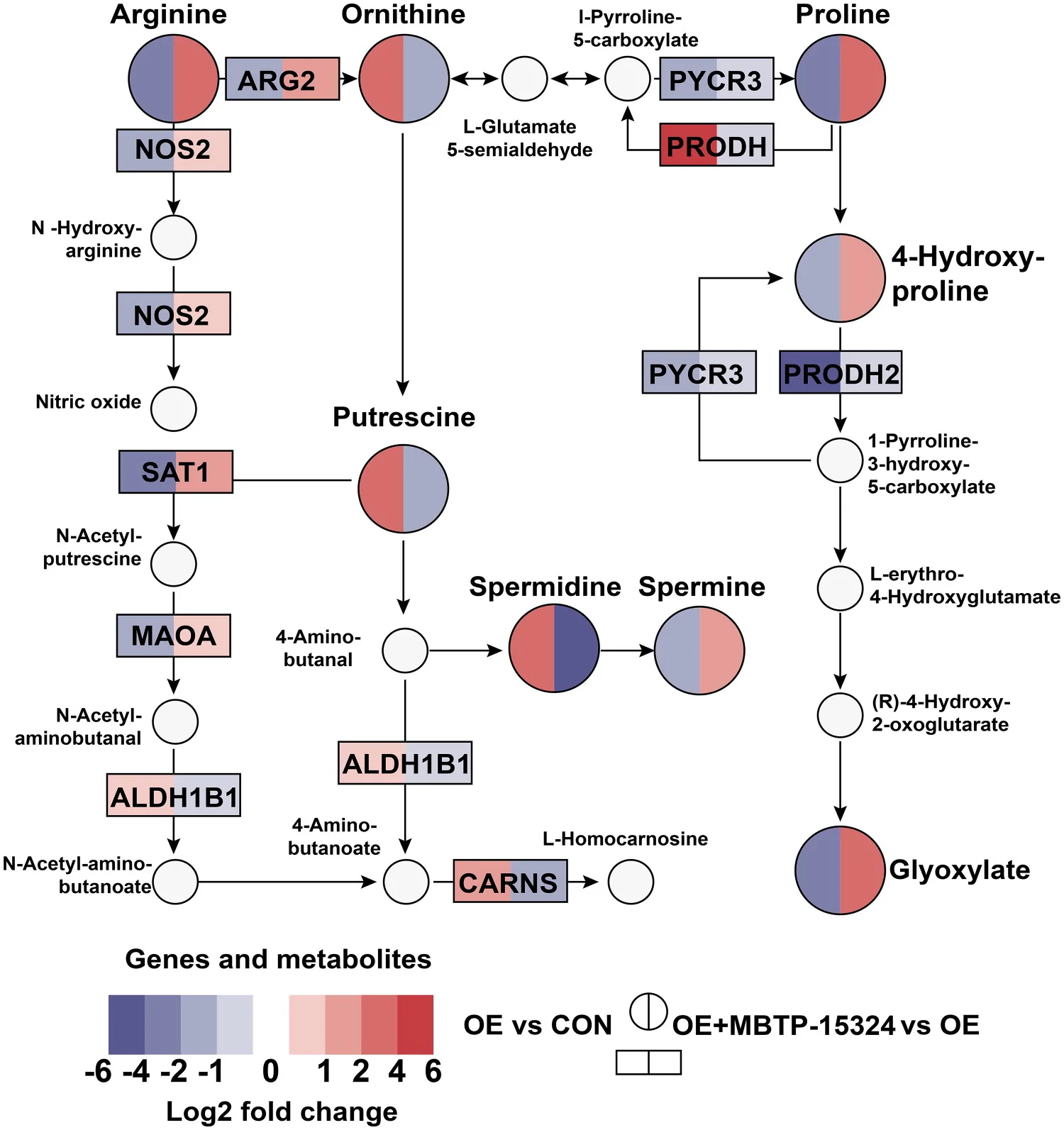
Integrative network analysis of transcriptomics and metabolomics reveals targeted remodelling of the arginine-proline metabolic axis by MBTP-15324. (A) Left half (OE vs. CON): Reflects the differences in transcript or metabolite abundance in the PRODH overexpression group (OE) compared to the wild-type control group (CON). (B) Right half (OE+MBTP-15324 vs. OE): Reflects the changes following MBTP-15324 drug intervention compared to the PRODH overexpression group, representing the rescue or reversal effect of the drug. This network graph integrates bulk RNA-seq and targeted metabolomics data to systematically illustrate the alteration patterns of key metabolic nodes in the arginine-proline and its downstream polyamine and hydroxyproline metabolic branches. Rectangular nodes represent metabolic enzyme genes (PRODH data were obtained via PCR analysis), and circular nodes represent metabolites. To visually compare changes before and after intervention, each node is split vertically into left and right halves.Node colours are mapped based on the Log_2_ fold change of differential expression: red shades indicate upregulation of gene expression or relative metabolite abundance (Log_2_FC > 0), while blue shades indicate significant downregulation (Log_2_FC < 0); colour depth is proportional to the magnitude of difference (see the bottom colour scale bar). (Data and abbreviation notes: Transcriptomics and metabolomics analyses were both based on independent biological replicate samples, n = 3 per group. CON, control group; OE, PRODH overexpression group.)

To further dissect the impacts of PRODH overexpression and MBTP-15324 intervention on the arginine-proline metabolic network, this study mapped differentially expressed genes and differential metabolites onto relevant metabolic pathways for integrated analysis (Figure 7). The results showed that PRODH overexpression significantly perturbed the metabolic network centred around arginine, ornithine, proline, and their downstream polyamine metabolites. Compared to the control group, proline and arginine levels were decreased in the PRODH-OE group, while metabolic nodes such as ornithine, putrescine, spermidine/spermine, and glyoxylate underwent distinct changes, indicating that PRODH overexpression induces pathway-associated metabolic perturbations in the arginine-proline network.

At the gene level, key metabolic enzymes in this pathway, including *ARG2*, *NOS2*, *SAT1*, *MAOA*, *ALDH1B1*, *PYCR3*, *PRODH*, *PRODH2*, and *CARNS*, exhibited differential expression, presenting good concordance with adjacent metabolite alterations. Notably, PRODH serves as a critical node for the conversion of proline to L-pyrroline-5-carboxylate, and its altered expression was consistent with the depletion of proline and the redistribution of downstream metabolites. Concurrently, *ARG2*-related ornithine production, *SAT1*/*MAOA*/*ALDH1B1*-related polyamine catabolism, and the *PYCR3*/*PRODH2*-related hydroxyproline metabolic branches were all impacted. This demonstrates that PRODH overexpression not only affects the metabolism of proline itself but also triggers synergistic alterations in the arginine, polyamine, and hydroxyproline metabolic branches (Supplementary Figure 4).

Following MBTP-15324 treatment, multiple metabolites and associated metabolic enzymes altered by PRODH overexpression exhibited a reverse regulatory trend. Specifically, proline and arginine levels showed a trend towards recovery, while the aberrant changes in ornithine, polyamine-related metabolites, and glyoxylate were mitigated to varying degrees. These results align with the aforementioned metabolomics analysis showing that MBTP-15324 restores proline levels and reduces metabolites such as succinate, ornithine, and glutathione, further indicating that MBTP-15324 inhibits the metabolic reprogramming mediated by PRODH overexpression (Supplementary Figure 5).

In summary, the pathway mapping results demonstrate that PRODH overexpression primarily impacts the arginine-proline metabolic axis, accompanied by a remodelling of the polyamine and hydroxyproline metabolic branches. MBTP-15324 intervention partially reverses these metabolic anomalies, indicating a distinct regulatory effect on the PRODH-associated metabolic network.

## Discussion

Focusing on PRODH-associated metabolic dependency and its potential allosteric regulatory strategies, this study identified the putative PRODH allosteric inhibitor MBTP-15324 and preliminarily evaluated its effects on metabolic reprogramming and ECM-related phenotypes in lung cancer cells. The main findings include: First, CavityPlus/CorrSite analysis suggested that Cavity1 exhibits a favourable balance among spatial accessibility, druggability score, and allosteric correlation with the orthosteric site, making it a suitable candidate allosteric pocket. Second, Hit-3/MBTP-15324, identified via virtual screening of the PMET library, generated a low-micromolar apparent binding response in the microscale thermophoresis (MST) assay within the PRODH-GFP fusion protein lysate system; the absence of a fittable concentration-dependent response in the GFP-only control supports that this signal primarily originates from PRODH-specific binding events. Third, molecular dynamics (MD) simulations supported that this candidate allosteric inhibitor maintains a relatively stable binding conformation within the predicted pocket of the 1TIW surrogate structural model. Fourth, MBTP-15324 attenuated cell viability, clonogenicity, and two-dimensional (2D) migration-related phenotypes in PRODH-overexpressing A549 and LLC cells. Fifth, transcriptomic and metabolomic analyses indicated that it can partially reverse arginine-proline metabolic dysregulation, as well as inflammation-, cell adhesion-, and ECM-related transcriptional programs in A549 PRODH-OE cells.

Regarding the proposed allosteric mechanism, the current computational pocket prediction and MD simulations provide theoretical evidence for the proposed binding mode within Cavity1, while the MST assay offers direct experimental validation of its apparent binding affinity in a PRODH-GFP lysate system. Together, the robust computational predictions, the low-micromolar experimental binding response, and the subsequent reversal of PRODH-associated cellular phenotypes collectively support a potential allosteric regulatory mechanism. However, it is important to distinguish that the precise allosteric binding site currently relies on computational prediction, and future high-resolution structural biology studies are required for direct experimental confirmation.

Mechanistically, PRODH overexpression was associated with reduced intracellular proline abundance and altered amino acid metabolic profiles, accompanied by elevations in metabolites such as succinate, ornithine, glutamine, and glutathione. This suggests that enhanced proline catabolism may further impact the tricarboxylic acid (TCA) cycle, redox homeostasis, and amino acid metabolic networks^21^. Following MBTP-15324 treatment, proline and arginine levels showed a trend towards recovery, whereas metabolites like succinate, ornithine, and glutathione decreased. Concurrently, ECM- and inflammation-related transcripts, such as *MMP10*, *CCL20*, and *CCL22*, were partially downregulated^22^. These results suggest that MBTP-15324 may influence the metabolism-transcription coupled network by modulating the PRODH overexpression-associated metabolic state, ultimately attenuating clonogenic and migration-related phenotypes in lung cancer cells. Because this study has not yet performed enzymatic inhibition assays with purified protein nor validated intracellular target occupancy, this mechanism should be appropriately defined as an explanatory model at the level of PRODH-associated phenotypes.

Compared with existing literature, the results of this study are consistent with reports that PRODH supports metabolic adaptation and malignant progression in specific tumour contexts. Previous studies have suggested that PRODH is involved in non-small cell lung cancer (NSCLC) progression, metastatic adaptation, and the regulation of proline metabolism^12^. This study further demonstrates, from the perspective of pharmacological intervention, that inhibiting the PRODH-associated metabolic state can attenuate cellular malignant phenotypes in PRODH-overexpressing lung cancer models. Concurrently, this study addresses shortcomings in current research on PRODH inhibitors. Previous chemical interventions have predominantly focused on the orthosteric substrate-binding site, whereas this study attempted to discover non-proline analogue scaffolds starting from a potential allosteric pocket, providing a novel approach for targeted PRODH regulation. It is important to note that the role of PRODH in tumours is context-dependent; therefore, the conclusions of this study are more applicable to PRODH-overexpressing or PRODH-dependent lung cancer cell models and cannot be simply extrapolated to all tumour types^9^^,^^23^.

The novelty of this study is primarily reflected in three aspects. Theoretically, it proposes that PRODH-associated proline catabolism may be coupled with ECM-associated gene-expression changes and inflammatory transcriptional programs, providing a new explanatory framework for understanding metabolic adaptation in lung cancer cells^24^. Methodologically, it established a comprehensive screening pipeline integrating pocket prediction, virtual screening, MST binding validation, MD simulations, cellular phenotyping, and multi-omics mechanistic analysis. In terms of research perspective, rather than being limited to the traditional orthosteric site, this study explored a candidate allosteric pocket and discovered MBTP-15324, which features a methyl benzoyl-thienyl-pyrrolone scaffold, thereby expanding the chemical space of PRODH-targeted molecules.This study holds both theoretical significance and practical application value. Theoretically, the results suggest that PRODH is not only a proline metabolic enzyme but also a crucial metabolic node linking amino acid metabolism, redox homeostasis, cell adhesion, and ECM phenotypes^25^. Practically, MBTP-15324 can serve as a candidate PRODH allosteric inhibitor, providing an allosteric inhibitor scaffold for metabolic interventions in lung cancer. A major strength of this study is its relatively complete chain of evidence, encompassing computational prediction, physical binding, dynamic stability, cellular phenotyping, and multi-omics validation, with relatively consistent phenotypic trends observed in both the A549 and LLC lung cancer cell models. Furthermore, the use of the GFP control helped exclude non-specific MST responses caused by the tag itself, enhancing the reliability of the binding results.

Nevertheless, this study has several areas for further refinement. First, limited by the availability of a high-resolution structure for full-length human PRODH, the computational screening and MD simulations utilised the *Escherichia coli* PutA PRODH domain as a surrogate model. While this provides a reference for candidate pocket identification, subsequent studies could further optimise the binding site analysis by incorporating human PRODH structural models and validating key residues. Second, although the MST results showed that MBTP-15324 exhibits a low-micromolar binding response in the PRODH-GFP lysate system without a binding signal in the GFP control, this Kd is more appropriately treated as an apparent affinity parameter. Future studies should further validate this through MST, surface plasmon resonance (SPR), differential scanning fluorimetry (DSF), and enzymatic activity assays using purified proteins. Finally, future efforts should evaluate the clinical application scope of PRODH as a therapeutic target and the feasibility of clinical development for the inhibitor MBTP-15324, promoting preclinical studies and clinical trials within a safe and effective range. Subsequent research could incorporate lung cancer models with varying PRODH expression levels and metabolic dependencies to clarify the applicable biomarkers, effective dose windows, and potential combination therapy strategies for MBTP-15324, thereby providing a more systematic experimental basis for its preclinical translation.

In conclusion, this study identified MBTP-15324 as a candidate PRODH allosteric inhibitor and preliminarily demonstrated its ability to modulate the arginine-proline metabolic network in PRODH-overexpressing lung cancer cells. MBTP-15324 attenuates proliferation- and migration-related phenotypes, and partially reverses inflammation and ECM-related transcriptional regulation^26^. This allosteric inhibitor provides a valuable scaffold for the subsequent development of PRODH-targeted therapeutics.

## Methods

### Prediction of candidate allosteric pockets in PRODH

Potential ligand-binding pockets on the surface of PRODH were predicted using the CavityPlus 2022 server^27^. Given the current lack of a high-resolution experimental structure for full-length human PRODH suitable for large-scale docking, the *Escherichia coli* PutA PRODH domain (PDB ID: 1TIW) was selected as a surrogate structural model in this study. Structural pre-processing included the removal of water molecules, hydrogen addition, and the completion of missing residues. The Cavity module was utilised to scan for cavities on the protein surface, and Cavity3, which contains catalysis-related residues such as MET-516, TYR-552, and ARG-458, was designated as the orthosteric reference site. Subsequently, the CorrSite module was employed under default parameters to evaluate the allosteric correlation between the other pockets and the orthosteric site. Based on a comprehensive evaluation of PredMaxpKd, PredAvepKd, DrugScore, CorrSiteScore, spatial openness, and docking feasibility, Cavity1 was selected as the candidate allosteric pocket for subsequent virtual screening^28^.

### Virtual screening

Structure-based virtual screening targeting the Cavity1 pocket was performed using Schrödinger Suite 2024–2. The protein was processed using the Protein Preparation Wizard to complete hydrogen addition, hydrogen bond network optimisation, energy minimisation, and state assignment at pH 7.4. The PMET (Proline Metabolism) small-molecule library, containing 1,033,000 compounds, was pre-processed with LigPrep and initially filtered according to Lipinski’s Rule of Five. The centre of the docking grid was set to X = −9.0, Y = 43.75, Z = 42.5, with dimensions of 16.0 × 13.5 × 15.0 Å. A hierarchical screening workflow was employed: High-Throughput Virtual Screening (HTVS) was first applied to retain the top 100 candidates, followed by Extra Precision (XP) flexible docking to narrow the pool down to 3 candidates. Prime MM-GBSA was then used to calculate the binding free energy, and QikProp was utilised to evaluate physicochemical properties and ADMET parameters. Protein-ligand interaction patterns were visualised using PyMOL 2.4.1. Integrating docking scores, MM-GBSA, ADMET profiles, MST affinities, and cellular phenotypic results, Hit-3 was identified as the primary putative PRODH allosteric inhibitor and designated as MBTP-15324. MBTP-15324 was purchased from TargetMol (Catalog No. 8018–8430; molecular formula: C_20_H_22_N_2_O_4_S; molecular weight: 386.47 g/mol) and dissolved in DMSO to prepare a 20 mM stock solution, which was stored at −80 °C.

### Molecular dynamics simulations

A 100-ns all-atom molecular dynamics (MD) simulation of the PRODH–FAD–MBTP-15324 complex was performed using GROMACS 2023.2. The initial system consisted of the PRODH surrogate structural model (PDB ID: 1TIW), the FAD cofactor, and the ligand Hit-3/MBTP-15324. The amber14SB_ROC_lipid17 force field was applied to the protein, while topologies for the small molecule and FAD were generated by Sobtop with GAFF parameters assigned. The TIP3P water model was used as the solvent. After solvation, Na^+^ and Cl^-^ ions were added to neutralise the system, and simulations were conducted under periodic boundary conditions. The system first underwent energy minimisation using the steepest descent method with a maximum of 50,000 steps and a convergence criterion of maximum force <1000 kJ·mol^−1^·nm^−1^. This was followed by 500 ps of NVT equilibration and 1000 ps of NPT equilibration at 300 K and 1.0 bar, during which position restraints were applied to the protein, FAD, and ligand. The production MD simulation was run for 100 ns without restraints at a step size of 2 fs. Temperature and pressure were controlled using the V-rescale thermostat and Parrinello-Rahman barostat, respectively. Long-range electrostatic interactions were computed using the Particle Mesh Ewald (PME) method, short-range interactions were truncated at 1.0 nm, and hydrogen bonds were constrained using the LINCS algorithm. Post-simulation analyses included RMSD, RMSF, Rg, hydrogen bonds, SASA, number of contacts within 0.35 nm, and Coul-SR/LJ-SR interaction energies. Binding free energy was calculated using an MM/PBSA script, and plots were generated using DuIvyTools^29^^,^^30^.

### Cell culture and stable cell line construction

HEK293T, LLC, and A549 cell lines were purchased from the Cell Bank of the Chinese Academy of Sciences or Xinsaimei (Cellcook). Cells were cultured in DMEM complete medium supplemented with 10% foetal bovine serum (FBS) and 1% penicillin-streptomycin at 37 °C in a 5% CO_2_ atmosphere. To establish stable PRODH-overexpressing cell lines, a target plasmid carrying the *mouse Prodh-3 × FLAG-CopGFP-Puro* expression cassette was co-transfected with psPAX2 and pMD2.G packaging plasmids into HEK293T cells. Viral supernatants were harvested at 48 h and 72 h post-transfection, filtered through a 0.45 μm membrane, and used to infect A549 or LLC cells for 24 h. Following infection, cells were subjected to continuous selection with 10 μg/mL puromycin until all uninfected control cells died. The surviving cells were expanded and used as stable PRODH-overexpressing (PRODH-OE) cell lines.

### Reverse transcription-quantitative PCR (RT-qPCR)

Total cellular RNA was extracted using an RNA extraction kit, and its concentration and purity were measured using a NanoDrop spectrophotometer. For each sample, 1,000 ng of RNA was reverse-transcribed using the PrimeScript RT Master Mix under conditions of 37 °C for 15 min and 85 °C for 15 s. The cDNA was diluted 5-fold and amplified on an ABI Real-Time PCR system using ChamQ Universal SYBR qPCR Master Mix. The thermocycling program consisted of an initial denaturation at 95 °C for 30 s, followed by 40 cycles of 95 °C for 10 s and 60 °C for 30 s, concluding with a melt curve analysis. Each sample was run in triplicate. The primer sequences were as follows: *PRODH*-F 5′-gcagagcacaaggagatgga-3′, *PRODH*-R 5′-tggtattgcttgtcccgctt-3′; *β-actin*-F 5′-tggcaccacaccttctacaa-3′, *β-actin*-R 5′-ccagaggcgtacagggatag-3′. Relative expression levels were calculated using the 2^-ΔΔCt method, with *β-actin* serving as the internal reference.

### Western blotting

Cells were washed with ice-cold PBS and lysed in RIPA buffer containing PMSF and phosphatase inhibitors on ice for 20 min. The lysates were centrifuged at 12,000 rpm for 10 min at 4 °C to collect the supernatant. Protein concentrations were determined using a BCA assay. Equal amounts of protein (20–40 μg per well) were separated by SDS-PAGE and transferred onto PVDF membranes. The membranes were blocked with 5% skim milk or 5% BSA for 2 h at room temperature, followed by overnight incubation at 4 °C with primary antibodies. The primary antibodies used included rabbit anti-PRODH (Proteintech, 22980–1-AP, 1:5000) and the internal control antibody anti-β-actin (Beyotime, AF2811, 1:1000) or anti-GAPDH (Servicebio, GB15002, 1:5000). After washing, the membranes were incubated with an HRP-conjugated secondary antibody (Xinsaimei, A0216, 1:1000) for 2 h at room temperature. Protein bands were visualised using an ECL reagent and captured using a Bio-Rad imaging system

### Binding affinity detection by microscale thermophoresis (MST)

The binding affinities between candidate compounds and PRODH were evaluated via MST using a NanoTemper Monolith NT.115 instrument, and the Kd values were fitted using MO Affinity Analysis v2.3 software. Cell lysates of HEK293T cells expressing the PRODH-GFP fusion protein served as the fluorescent target, while GFP-only lysates were used as the tag background control. The assay buffer used was TNT buffer (10 mM Tris-HCl, 150 mM NaCl, 0.05% Tween 20, pH 7.8–8.2). Following preliminary tests confirming no significant capillary adsorption or sample aggregation, the PRODH-GFP lysates and GFP-only controls were diluted 1:5 and 1:10, respectively. Hit-1, Hit-2, and Hit-3/MBTP-15324 were subjected to a 16-point 2-fold serial dilution in TNT buffer containing 5% DMSO, yielding detection ranges of 400–0.0122 μM, 50–0.00153 μM, and 25–0.000763 μM, respectively. The ligands and lysates were mixed at a 1:1 ratio and incubated at room temperature for 15 min prior to sample loading. Detection parameters were set to Nano-BLUE excitation, Excitation Power 20%, MST Power 40%, and a temperature of 25.0 °C. At least three independent pipetting and measurement replicates were performed for each compound^31^.

### Cell viability assays and phenotypic experiments

Cell viability was assessed using the CCK-8 assay. Cells were seeded into 96-well plates at a density of 2 × 10³ cells/well, with three replicate wells per group. After adhering overnight, cells were treated with 0.1–100 μM MBTP-15324 or an equal volume of DMSO. Following treatment, 10 μL of CCK-8 reagent was added to each well, incubated for 1.5–2 h, and the absorbance was measured at 450 nm. For the time-dependent assay, cells were treated with 50 μM MBTP-15324 and evaluated at 0, 24, 48, and 72 h. In the colony formation assay, cells were seeded into 6-well plates at 500 cells/well and continuously treated with 50 μM MBTP-15324 for 10–14 days, with the medium replaced every 2–3 days. The colonies were then fixed with 4% paraformaldehyde for 20 min, stained with 0.5% crystal violet for 20 min, and the colony area was quantified using ImageJ 1.54. For the wound healing assay, cells were grown to 90%-100% confluence, and a scratch was created using a 200 μL pipette tip. After washing with PBS, the medium was replaced with medium containing 1% FBS and 50 μM MBTP-15324. Images were captured at 0 h and 24 h, and the healing rate was calculated based on the change in the wound area. All phenotypic experiments were independently repeated at least three times.

### Bulk RNA-seq and bioinformatic analysis

A549 cells from the Control, PRODH-OE, and PRODH-OE+MBTP-15324 groups were collected, with three biological replicates per group. The drug group was treated with 50 μM MBTP-15324 for 24 h, while the control group received an equal volume of DMSO. Total RNA was extracted using TRIzol reagent and quality-checked using a NanoDrop and an Agilent 2100 Bioanalyzer. Samples proceeding to library construction met the criteria of RIN ≥ 7.0, A260/A280 = 1.8–2.1, and A260/A230 ≥ 1.8. Poly(A)+ mRNA libraries were constructed from 1,000 ng of RNA per sample using the VAHTS Universal V6 RNA-seq Library Prep Kit for Illumina, followed by 150 bp paired-end sequencing on the Illumina NovaSeq 6000 platform. Raw reads underwent quality control via FastQC v0.11.9 and trimming via Trimmomatic v0.39, and were subsequently mapped to the GRCh38/hg38 reference genome using HISAT2 v2.2.1. Expression levels were estimated using StringTie v2.1.7 and featureCounts v2.0.1. Differential expression analysis was performed in R using DESeq2 v1.38.3, with thresholds set at |log2FC| > 1 and adjusted *p* values < 0.05. PCA, volcano plots, heatmaps, GO/KEGG, and GSEA analyses were executed using ggplot2, pheatmap, clusterProfiler v4.6.2, GSEA v4.3.2, and MSigDB v7.5.1, respectively^32^^,^^33^.

### Targeted metabolomics analysis

To evaluate the impact of PRODH overexpression and MBTP-15324 intervention on the cellular metabolic state, A549 cells from the CON, PRODH-OE, and OE+MBTP-15324 groups were harvested (three biological replicates per group). The drug group was treated with 50 μM MBTP-15324 for 24 h, while the control group received an equal volume of DMSO. For each sample, 2 × 10^6^ cells were washed with ice-cold PBS, and metabolites were extracted using 1,000 μL of ice-cold 80% MS-grade methanol. The samples were vortexed and centrifuged at 14,000 × g for 15 min. The supernatant was collected, vacuum-dried, and reconstituted in 50 μL of methanol-water (90:10, v/v). A 10 μL aliquot of each sample was injected into an Agilent 6490 Triple Quadrupole LC-MS/MS system. Liquid chromatography separation was achieved using a Kinetex 2.6 μm PS C18 column (150 × 2.1 mm). Mass spectrometry was performed in Multiple Reaction Monitoring (MRM) mode. Target metabolites were confirmed based on the retention times, MRM transitions, and MS responses of purified standards. Peak areas were integrated using MassHunter Qualitative Analysis Software B.06.00.633.0 and corrected against the initial cell count and batch quality control (QC) samples. Normalised data underwent log2 transformation and standardisation before performing PCA, clustering, heatmaps, volcano plots, correlation, and pathway analyses using MetaboAnalyst 5.0 and R. The thresholds for screening differential metabolites were Variable Importance in Projection (VIP) > 1 and *p* < 0.05. Differentially expressed genes and differential metabolites were further mapped onto arginine-proline metabolism-related pathways annotated by KEGG and HMDB^34^.

### Statistical analyses

Statistical analyses were performed using GraphPad Prism 9.0. Quantitative data are presented as mean ± SEM. Unless otherwise stated, each experimental group included at least three independent biological replicates, and no data was excluded. Comparisons between two groups were conducted using an unpaired two-tailed Student’s t-test. Comparisons among multiple groups were analysed using one-way or two-way ANOVA followed by Tukey’s *post hoc* test for multiple comparisons correction. Data that did not meet the assumptions of parametric tests were analysed using the two-tailed Mann-Whitney rank-sum test or ANOVA on ranks. Relative expression or relative abundance data were normalised to the respective control group prior to statistical analysis. For omics data, multiple testing corrections were applied using the Benjamini-Hochberg procedure, reporting the adjusted *p* values or False Discovery Rate (FDR). *p* < 0.05 was considered statistically significant. Significance is denoted as *p < 0.05, *p* < 0.01, ***p < 0.001, and ****p < 0.0001; NS indicates no statistical significance.

## Supplementary Material

Suppl Fig 2.pdf

Suppl Fig 3.pdf

Suppl Fig 4.pdf

Suppl Fig 5.pdf

Suppl Fig 1.pdf

## Data Availability

The raw RNA-seq data generated in this study will be deposited in a public repository prior to publication. During the review process, these datasets, along with all other data supporting the findings of this study, are available from the corresponding author upon reasonable request.

## References

[1] Liu Y, Mao C, Wang M, Liu N, Ouyang L, Liu S, Tang H, Cao Y, Liu S, Wang X, et al. Cancer progression is mediated by proline catabolism in non-small cell lung cancer. Oncogene. 2020;39(11):2358–2376. 31911619 10.1038/s41388-019-1151-5

[2] Bray F, Laversanne M, Sung H, Ferlay J, Siegel RL, Soerjomataram I, Jemal A. Global cancer statistics 2022: GLOBOCAN estimates of incidence and mortality worldwide for 36 cancers in 185 countries. CA Cancer J Clin. 2024;74(3):229–263. 38572751 10.3322/caac.21834

[3] Pavlova NN, Zhu J, Thompson CB. The hallmarks of cancer metabolism: still emerging. Cell Metab. 2022;34(3):355–377. 35123658 10.1016/j.cmet.2022.01.007 PMC8891094

[4] Dong S, Li T. Metabolic reprogramming in cancer: signaling pathways and therapeutic targets. Mol Cancer. 2026;25(1):60. 10.1186/s12943-026-02582-0. PMC12967016 41634663

[5] Karlstaedt A, Barrett M, Hu R, Gammons ST, Ky B. Cardio-oncology: understanding the intersections between cardiac metabolism and cancer biology. JACC Basic Transl Sci. 2021;6(8):705–718. 34466757 10.1016/j.jacbts.2021.05.008 PMC8385559

[6] Galli U, Travelli C, Massarotti A, Fakhfouri G, Rahimian R, Tron GC, Genazzani AA. Medicinal chemistry of nicotinamide phosphoribosyltransferase (NAMPT) inhibitors. J Med Chem. 2013;56(16):6279–6296. 23679915 10.1021/jm4001049

[7] Phang JM. Proline Metabolism in cell regulation and cancer biology: recent advances and hypotheses. Antioxid Redox Signal. 2019;30(4):635–649. 28990419 10.1089/ars.2017.7350 PMC6338564

[8] Wang D, Duan JJ, Guo YF, Chen JJ, Chen TQ, Wang J, Yu SC. Targeting the glutamine-arginine-proline metabolism axis in cancer. J Enzyme Inhib Med Chem. 2024;39(1):2367129. 39051546 10.1080/14756366.2024.2367129 PMC11275534

[9] Liu W, Phang JM. Proline dehydrogenase (oxidase), a mitochondrial tumor suppressor, and autophagy under the hypoxia microenvironment. Autophagy. 2012;8(9):1407–1409. 22885468 10.4161/auto.21152 PMC3442893

[10] Liu Y, Borchert GL, Donald SP, A Diwan B, Anver M, Phang JM. Proline oxidase functions as a mitochondrial tumor suppressor in human cancers. Cancer Res. 2009;69(16):6414–6422. 19654292 10.1158/0008-5472.CAN-09-1223 PMC4287397

[11] Xu X, Zhang G, Chen Y, Xu W, Liu Y, Ji G, Xu H. Can proline dehydrogenase-a key enzyme involved in proline metabolism-be a novel target for cancer therapy? Front Oncol. 2023;13:1254439. 38023181 10.3389/fonc.2023.1254439 PMC10661406

[12] Elia I, Broekaert D, Christen S, Boon R, Radaelli E, Orth MF, Verfaillie C, Grünewald TGP, Fendt SM. Proline metabolism supports metastasis formation and could be inhibited to selectively target metastasizing cancer cells. Nat Commun. 2017;8(1):15267. 28492237 10.1038/ncomms15267 PMC5437289

[13] Tanner JJ, Fendt SM, Becker DF. The proline cycle as a potential cancer therapy target. Biochemistry. 2018;57(25):3433–3444. 29648801 10.1021/acs.biochem.8b00215 PMC6026536

[14] Scott GK, Yau C, Becker BC, Khateeb S, Mahoney S, Jensen MB, Hann B, Cowen BJ, Pegan SD, Benz CC. Targeting mitochondrial proline dehydrogenase with a suicide inhibitor to exploit synthetic lethal interactions with p53 upregulation and glutaminase inhibition. Mol Cancer Ther. 2019;18(8):1374–1385. 31189611 10.1158/1535-7163.MCT-18-1323 PMC6679736

[15] Jones S, Ahmet J, Ayton K, Ball M, Cockerill M, Fairweather E, Hamilton N, Harper P, Hitchin J, Jordan A, et al. Discovery and optimization of allosteric inhibitors of mutant isocitrate dehydrogenase 1 (R132H IDH1) displaying activity in human acute myeloid leukemia cells. J Med Chem. 2016;59(24):11120–11137. 28002956 10.1021/acs.jmedchem.6b01320

[16] Mai TT, Lai NV, Lam TP, Truong LN, Nguyen MN, Nguyen NV, Phan MH, Tuong LT, Thai KM. Validated virtual screening models for identifying allosteric inhibitors of ATP-citrate lyase: the role of docking scores and protein-ligand interaction similarity in hit selection. Mol Divers. 2026;30(4):6177–6192. 41865345 10.1007/s11030-026-11519-0

[17] Li C, Yang Q, Zhang L. Identification of putative allosteric inhibitors of BCKDK via virtual screening and biological evaluation. J Enzyme Inhib Med Chem. 2024;39(1):2290458. 38059302 10.1080/14756366.2023.2290458 PMC11721764

[18] Ye X, Deng S, Luo D, Fu W, Du Q, Xue W. Discovery of a new allosteric inhibitor for human dopamine transporter by physics-based modeling and experimental validation. Eur J Med Chem. 2026;303:118403. 41330154 10.1016/j.ejmech.2025.118403

[19] Bogner AN, Ji J, Tanner JJ. Structure-based engineering of minimal proline dehydrogenase domains for inhibitor discovery. Protein Eng Des Sel. 2022;35:gzac016. 10.1093/protein/gzac016. PMC9801229 36448708

[20] Zhou Y, Li C, Bai D, Jing L, Han L, Guo C, Yang Q. Proline metabolism reprogramming in cancer reveals the regulation of PRODH/POX as target. Biomed Pharmacother. 2026;195:119040. 41604890 10.1016/j.biopha.2026.119040

[21] Selak MA, Armour SM, MacKenzie ED, Boulahbel H, Watson DG, Mansfield KD, Pan Y, Simon MC, Thompson CB, Gottlieb E. Succinate links TCA cycle dysfunction to oncogenesis by inhibiting HIF-alpha prolyl hydroxylase. Cancer Cell. 2005;7(1):77–85. 15652751 10.1016/j.ccr.2004.11.022

[22] Justilien V, Regala RP, Tseng IC, Walsh MP, Batra J, Radisky ES, Murray NR, Fields AP. Matrix metalloproteinase-10 is required for lung cancer stem cell maintenance, tumor initiation and metastatic potential. PLoS One. 2012;7(4):e35040. 22545096 10.1371/journal.pone.0035040 PMC3335833

[23] Pandhare J, Donald SP, Cooper SK, Phang JM. Regulation and function of proline oxidase under nutrient stress. J Cell Biochem. 2009;107(4):759–768. 19415679 10.1002/jcb.22174 PMC2801574

[24] Cui C, Wang J, Guo L, Wu C. PINCH-1 promotes Δ(1)-pyrroline-5-carboxylate synthase expression and contributes to proline metabolic reprogramming in lung adenocarcinoma. Amino Acids. 2021;53(12):1875–1890. 34283311 10.1007/s00726-021-03050-3

[25] Liu W, Phang JM. Proline dehydrogenase (oxidase) in cancer. Biofactors. 2012;38(6):398–406. 22886911 10.1002/biof.1036 PMC7479541

[26] Lee S, Nakamura E, Yang H, Wei W, Linggi MS, Sajan MP, Farese RV, Freeman RS, Carter BD, Kaelin WG, Jr., et al. Neuronal apoptosis linked to EglN3 prolyl hydroxylase and familial pheochromocytoma genes: developmental culling and cancer. Cancer Cell. 2005;8(2):155–167. 16098468 10.1016/j.ccr.2005.06.015

[27] Wang S, Xie J, Pei J, Lai L. CavityPlus 2022 update: an integrated platform for comprehensive protein cavity detection and property analyses with user-friendly tools and cavity databases. J Mol Biol. 2023;435(14):168141. 37356903 10.1016/j.jmb.2023.168141

[28] Xu Y, Wang S, Hu Q, Gao S, Ma X, Zhang W, Shen Y, Chen F, Lai L, Pei J. CavityPlus: a web server for protein cavity detection with pharmacophore modelling, allosteric site identification and covalent ligand binding ability prediction. Nucleic Acids Res. 2018;46(W1):W374–w379. 29750256 10.1093/nar/gky380 PMC6031032

[29] Pronk S, Páll S, Schulz R, Larsson P, Bjelkmar P, Apostolov R, Shirts MR, Smith JC, Kasson PM, van der Spoel D, et al. GROMACS 4.5: a high-throughput and highly parallel open source molecular simulation toolkit. Bioinformatics. 2013;29(7):845–854. 23407358 10.1093/bioinformatics/btt055 PMC3605599

[30] Maier JA, Martinez C, Kasavajhala K, Wickstrom L, Hauser KE, Simmerling C. ff14SB: improving the accuracy of protein side chain and backbone parameters from ff99SB. J Chem Theory Comput. 2015;11(8):3696–3713. 26574453 10.1021/acs.jctc.5b00255 PMC4821407

[31] Wienken CJ, Baaske P, Rothbauer U, Braun D, Duhr S. Protein-binding assays in biological liquids using microscale thermophoresis. Nat Commun. 2010;1(1):100. 20981028 10.1038/ncomms1093

[32] Love MI, Huber W, Anders S. Moderated estimation of fold change and dispersion for RNA-seq data with DESeq2. Genome Biol. 2014;15(12):550. 25516281 10.1186/s13059-014-0550-8 PMC4302049

[33] Wu T, Hu E, Xu S, Chen M, Guo P, Dai Z, Feng T, Zhou L, Tang W, Zhan L, et al. clusterProfiler 4.0: A universal enrichment tool for interpreting omics data. Innovation (Camb). 2021;2(3):100141. 34557778 10.1016/j.xinn.2021.100141 PMC8454663

[34] Pang Z, Chong J, Zhou G, de Lima Morais DA, Chang L, Barrette M, Gauthier C, Jacques P, Li S, Xia J. MetaboAnalyst 5.0: narrowing the gap between raw spectra and functional insights. Nucleic Acids Res. 2021;49(W1):W388–w396. 34019663 10.1093/nar/gkab382 PMC8265181

