# Supplementary material for: Identification of a candidate PRODH allosteric inhibitor MBTP-15324: modulation of proline metabolite profiles and ECM-associated gene-expression changes in lung cancer cells
Source: J Enzyme Inhib Med Chem. 2026 Aug 24;41(1):2719719. doi: 10.1080/14756366.2026.2719719 (PMC13508488; doi:10.1080/14756366.2026.2719719)
Supplement: Supplementary file 1 [file IENZ_A_2719719_SM9148.pdf]

Suppl. Fig. 1

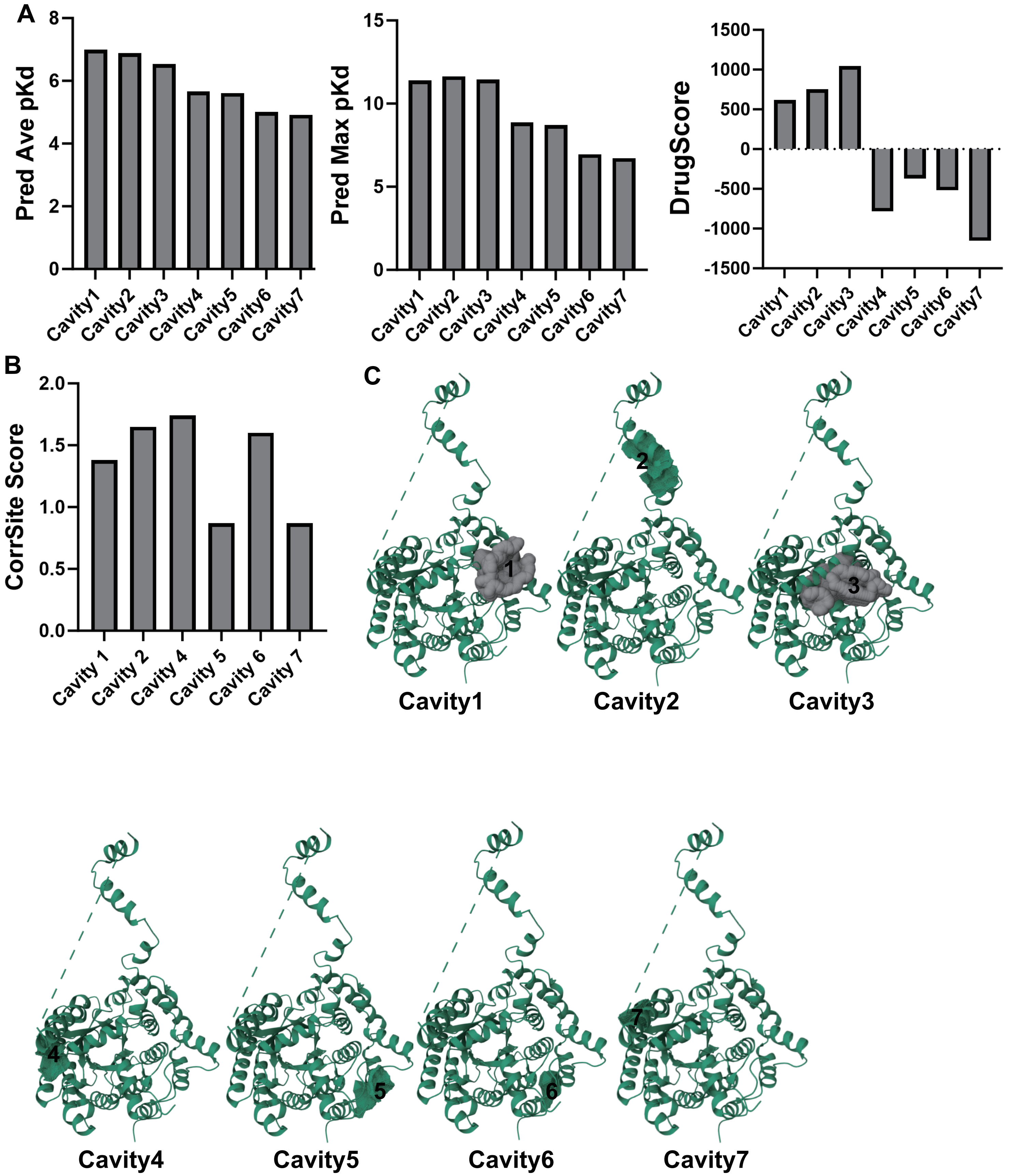
A
Pred Ave pKd
0
2
4
6
8
Cavity1
Cavity2
Cavity3
Cavity4
Cavity5
Cavity6
Cavity7
Pred Max pKd
0
5
10
15
DrugScore
1500
1000
500
0
-500
-1000
-1500
B
CorrSite Score
0.0
0.5
1.0
1.5
2.0
Cavity 1
Cavity 2
Cavity 4
Cavity 5
Cavity 6
Cavity 7
C
1
2
3
4
5
6
7
Cavity1
Cavity2
Cavity3
Cavity4
Cavity5
Cavity6
Cavity7
