# Supplementary material for: Identification of a candidate PRODH allosteric inhibitor MBTP-15324: modulation of proline metabolite profiles and ECM-associated gene-expression changes in lung cancer cells
Source: J Enzyme Inhib Med Chem. 2026 Aug 24;41(1):2719719. doi: 10.1080/14756366.2026.2719719 (PMC13508488; doi:10.1080/14756366.2026.2719719)
Supplement: Supplementary file 2 [file IENZ_A_2719719_SM9149.pdf]

Suppl Fig 5

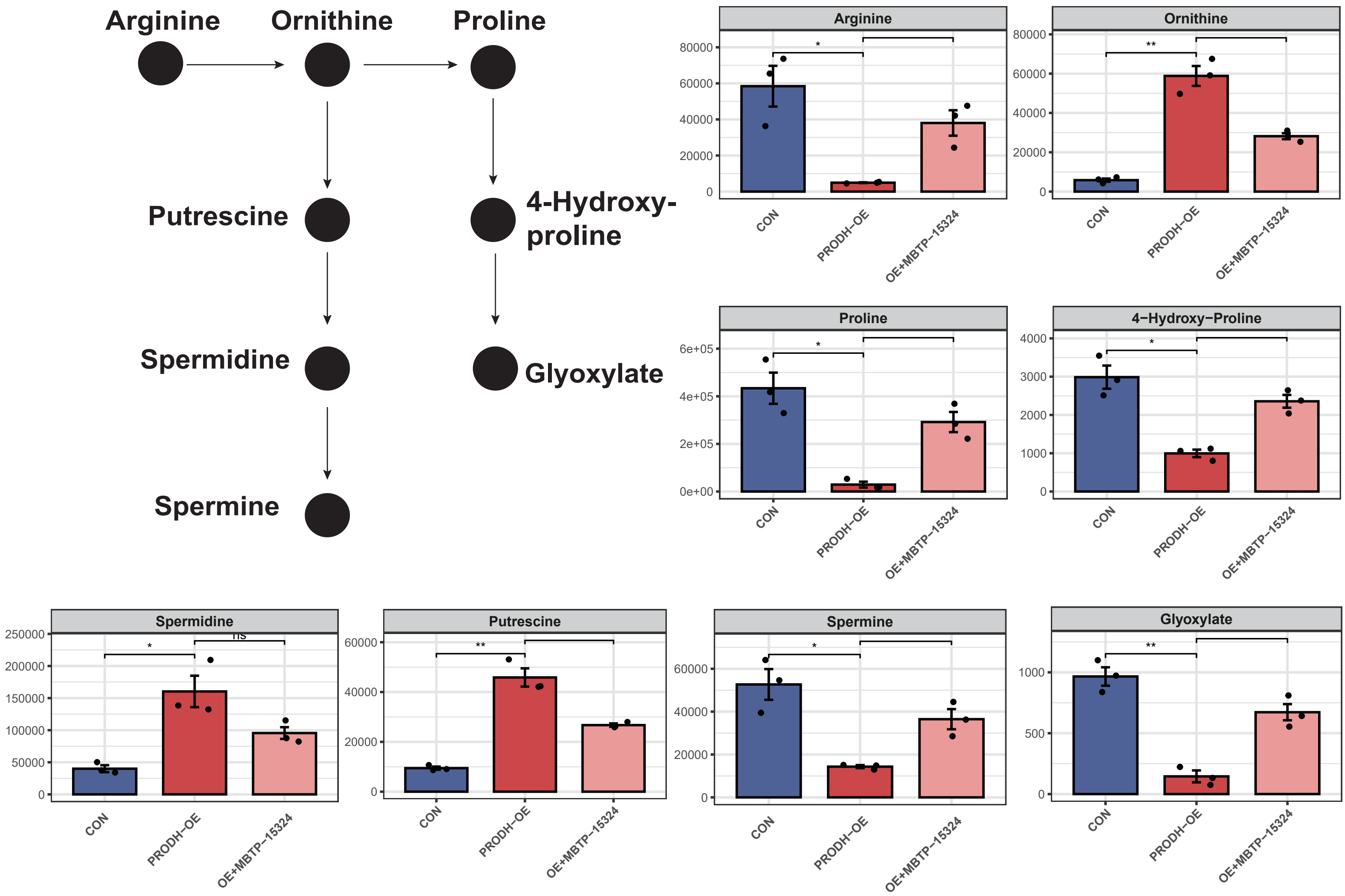
Arginine
Ornithine
Proline
Putrescine
4-Hydroxy-proline
Spermidine
Glyoxylate
Spermine
Arginine
Ornithine
Proline
4−Hydroxy−Proline
Spermidine
Putrescine
Spermine
Glyoxylate
ns
CON
PRODH−OE
OE+MBTP−15324
