# Supplementary material for: Identification of a candidate PRODH allosteric inhibitor MBTP-15324: modulation of proline metabolite profiles and ECM-associated gene-expression changes in lung cancer cells
Source: J Enzyme Inhib Med Chem. 2026 Aug 24;41(1):2719719. doi: 10.1080/14756366.2026.2719719 (PMC13508488; doi:10.1080/14756366.2026.2719719)
Supplement: Supplementary file 3 [file IENZ_A_2719719_SM9150.pdf]

Suppl. Fig. 4

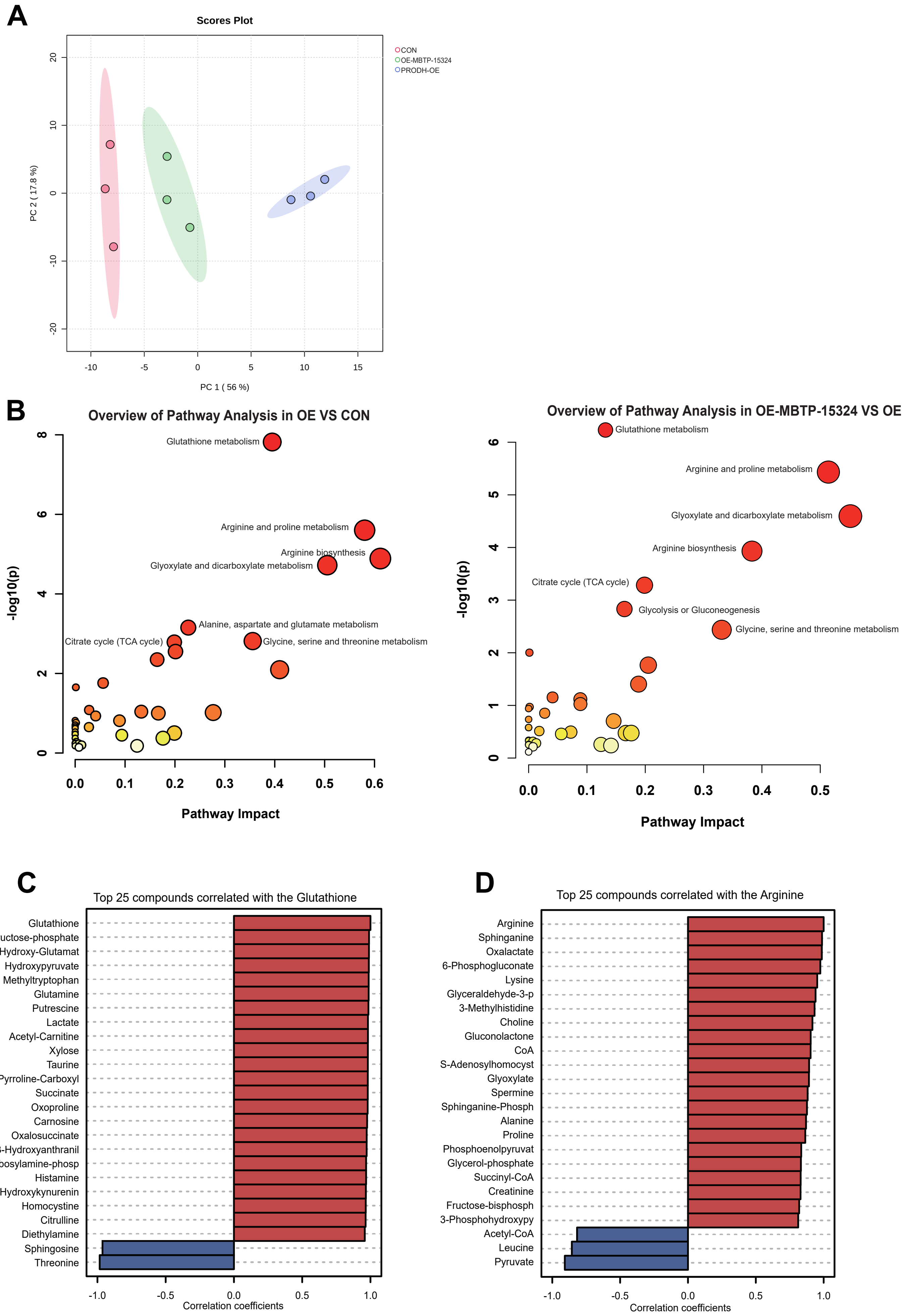

A
Scores Plot
CON
OE-MBTP-15324
PRODH-OE
PC 1 (56 %)
PC 2 (17.8 %)
B
Overview of Pathway Analysis in OE VS CON
Glutathione metabolism
Arginine and proline metabolism
Arginine biosynthesis
Glyoxylate and dicarboxylate metabolism
Alanine, aspartate and glutamate metabolism
Citrate cycle (TCA cycle)
Glycine, serine and threonine metabolism
-log10(p)
Pathway Impact
Overview of Pathway Analysis in OE-MBTP-15324 VS OE
Glutathione metabolism
Arginine and proline metabolism
Glyoxylate and dicarboxylate metabolism
Arginine biosynthesis
Citrate cycle (TCA cycle)
Glycolysis or Gluconeogenesis
Glycine, serine and threonine metabolism
-log10(p)
Pathway Impact
C
Top 25 compounds correlated with the Glutathione
Glutathione
Fructose-phosphate
4-Hydroxy-Glutamat
Hydroxypyruvate
Methyltryptophan
Glutamine
Putrescine
Lactate
Acetyl-Carnitine
Xylose
Taurine
Pyrroline-Carboxyl
Succinate
Oxoproline
Carnosine
Oxalosuccinate
3-Hydroxyanthranil
Ribosylamine-phosp
Histamine
3-Hydroxykynurenin
Homocystine
Citrulline
Diethylamine
Sphingosine
Threonine
Correlation coefficients
D
Top 25 compounds correlated with the Arginine
Arginine
Sphinganine
Oxalactate
6-Phosphogluconate
Lysine
Glyceraldehyde-3-p
3-Methylhistidine
Choline
Gluconolactone
CoA
S-Adenosylhomocyst
Glyoxylate
Spermine
Sphinganine-Phosph
Alanine
Proline
Phosphoenolpyruvat
Glycerol-phosphate
Succinyl-CoA
Creatinine
Fructose-bisphosph
3-Phosphohydroxypy
Acetyl-CoA
Leucine
Pyruvate
Correlation coefficients
