# Supplementary material for: Identification of a candidate PRODH allosteric inhibitor MBTP-15324: modulation of proline metabolite profiles and ECM-associated gene-expression changes in lung cancer cells
Source: J Enzyme Inhib Med Chem. 2026 Aug 24;41(1):2719719. doi: 10.1080/14756366.2026.2719719 (PMC13508488; doi:10.1080/14756366.2026.2719719)

Suppl. Fig. 2

A

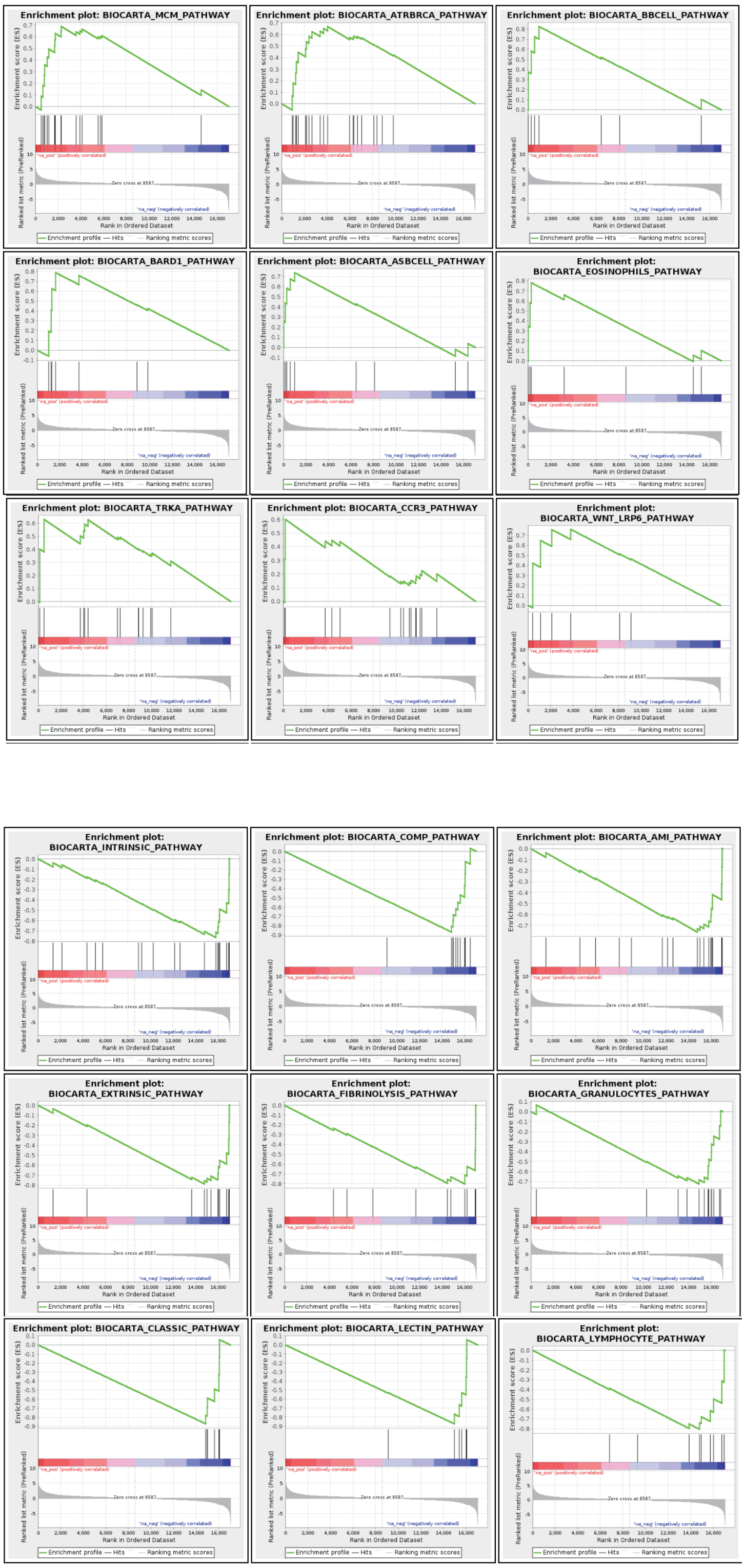

B

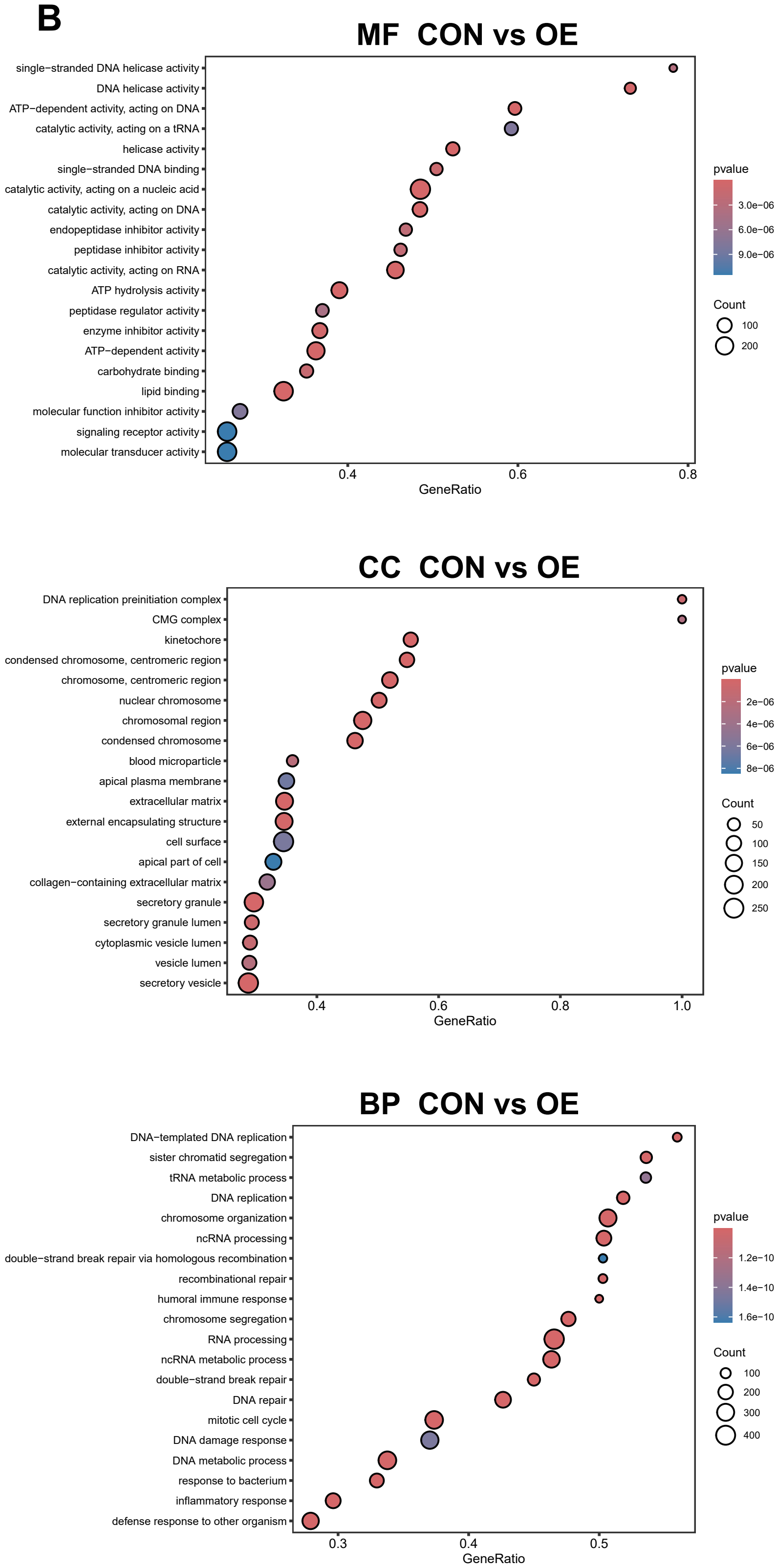

Supplement: Supplementary file 5 [file IENZ_A_2719719_SM9152.pdf]
